# Draft Sequencing of the Heterozygous Diploid Genome of Satsuma (*Citrus unshiu* Marc.) Using a Hybrid Assembly Approach

**DOI:** 10.3389/fgene.2017.00180

**Published:** 2017-12-05

**Authors:** Tokurou Shimizu, Yasuhiro Tanizawa, Takako Mochizuki, Hideki Nagasaki, Terutaka Yoshioka, Atsushi Toyoda, Asao Fujiyama, Eli Kaminuma, Yasukazu Nakamura

**Affiliations:** ^1^Division of Citrus Research, Institute of Fruit Tree and Tea Science, National Agriculture and Food Research Organization, Shimizu, Japan; ^2^Genome Informatics Laboratory, Center for Information Biology, National Institute of Genetics, Mishima, Japan; ^3^Comparative Genomics Laboratory, Center for Information Biology, National Institute of Genetics, Mishima, Japan

**Keywords:** citrus, Satsuma, draft genome assembly, gene prediction, genome synteny, gibberellic acid biosynthesis, carotenoid biosynthesis, parentage analysis

## Abstract

Satsuma (*Citrus unshiu* Marc.) is one of the most abundantly produced mandarin varieties of citrus, known for its seedless fruit production and as a breeding parent of citrus. *De novo* assembly of the heterozygous diploid genome of Satsuma (“Miyagawa Wase”) was conducted by a hybrid assembly approach using short-read sequences, three mate-pair libraries, and a long-read sequence of PacBio by the PLATANUS assembler. The assembled sequence, with a total size of 359.7 Mb at the N_50_ length of 386,404 bp, consisted of 20,876 scaffolds. Pseudomolecules of Satsuma constructed by aligning the scaffolds to three genetic maps showed genome-wide synteny to the genomes of Clementine, pummelo, and sweet orange. Gene prediction by modeling with MAKER-P proposed 29,024 genes and 37,970 mRNA; additionally, gene prediction analysis found candidates for novel genes in several biosynthesis pathways for gibberellin and violaxanthin catabolism. BUSCO scores for the assembled scaffold and predicted transcripts, and another analysis by BAC end sequence mapping indicated the assembled genome consistency was close to those of the haploid Clementine, pummel, and sweet orange genomes. The number of repeat elements and long terminal repeat retrotransposon were comparable to those of the seven citrus genomes; this suggested no significant failure in the assembly at the repeat region. A resequencing application using the assembled sequence confirmed that both kunenbo-A and Satsuma are offsprings of Kishu, and Satsuma is a back-crossed offspring of Kishu. These results illustrated the performance of the hybrid assembly approach and its ability to construct an accurate heterozygous diploid genome.

## Introduction

Genome sequencing of various citrus genomes has contributed to gene prediction, DNA marker development and the elucidation of the phylogenetic origin and domestication process of *Citrus* (Gmitter et al., 2012; Xu et al., 2013; Wu et al., 2014). Because of the economic and horticultural importance of citrus production, which exceeded 121 million tons worldwide during 2013–2014 (Intergovernmental Group on Citrus Fruits, 2016), genome sequences are anticipated to promote genome-wide association studies (GWAS) and genomic selection (GS) in citrus breeding (Minamikawa et al., 2017), thus, facilitating the development of promising varieties and improvement of tolerance to the devastating citrus greening disease (Huanglongbing, HLB) (van Nocker and Gardiner, 2014). Satsuma (or Satsuma mandarin, *Citrus unshiu* Marc.), one of the most produced mandarin varieties worldwide, produces practically seedless fruit by strong male-female sterility in combination with parthenocarpy (Tanaka, 1932). A recent phylogenetic study of Satsuma with DNA markers using a statistical genetic approach revealed that it is a backcrossed offspring (BC_1_) of the mandarin variety Kishu (*C. kinokuni* hort. ex Tanaka) (Shimizu et al., 2016b). While many mutant strains have been discovered as sports of Satsuma or selected from nucellar seedlings (Nishiura, 1964), it has been used as a breeding parent in several countries -including Japan -to develop some key hybrid varieties, such as Kiyomi (Nishiura et al., 1983), Harehime (Yoshida et al., 2005) and Kara (Hodgson, 1967). Furthermore, recent functional analysis uncovered the beneficial health effects of β-cryptoxanthin, which is particularly abundant in Satsuma fruits (Sugiura et al., 2015).

The explosive growth of next-generation sequencing technologies enabled whole-genome sequence assembly in fruit tree varieties; thereby allowing genome sequences for major fruit tree varieties of papaya (Ming et al., 2008), apple (Velasco et al., 2010; Daccord et al., 2017), grapevine (Velasco et al., 2007) and peach (International Peach Genome Initiative et al., 2013) to be released. However, high-quality genome assembly in fruit tree varieties is a difficult task because of their higher heterozygosity. Therefore, haploid plants or doubled haploid plants have been used in many genome-sequencing projects. In the case of citruses, most varieties are diploids of small genome size of 360–380 Mb (Gmitter et al., 2012), but many —except for particular pomelo varieties— exhibit high heterozygosity (in Satsuma, *H*o = 0.435) (Shimizu et al., 2016a), because of their hybrid origin (Shimizu et al., 2016b). Initial attempts to build the reference genome sequences of citrus varieties selected doubled haploid or haploid plants for their reference samples to reduce redundancy for better assembly. Accordingly, Huazhong Agricultural University in China released the draft genome assembly of sweet orange [*C. sinensis* (L.) Osbeck] with 4,811 scaffolds and total contig sequence length of 321 Mb (Xu et al., 2013). Another genome sequencing project led by the International Citrus Genome Consortium (ICGC: http://www.citrusgenome.ucr.edu/) generated a high-quality draft assembly of a haploid Clementine (*C. clementina* hort. ex Tanaka), which consists of 1,398 scaffolds with a total length of 301 Mb (Wu et al., 2014). Recently, Huazhong Agricultural University group released the latest assembly of haploid pummelo (*C. maxima* Merr.) and doubled haploid sweet orange genomes with three draft genomes of papeda, citron, and Atalantia (Wang et al., 2017). Sequencing a haploid plant is a secure approach to building high-quality genome sequences, whereas any side effects during haploid plant development on the sequence of the genome are not evident. Furthermore, continued efforts will be required to obtain a haploid plant, as suggested by the repeated failed attempts with many varieties in the past (Germana, 2007). Consequently, constructing genome sequences directly for various heterozygous citrus varieties is anticipated to expand the comparative genomic study among them and improve DNA marker analysis for marker-assisted selection (MAS) and novel gene discovery.

In this study, a *de novo* assembly of the heterozygous diploid genome of Satsuma was conducted from short-read sequences by an assembler focused on the heterozygous genome (Kajitani et al., 2014). This assembly approach demonstrated its usefulness during the heterozygous genome assembly of a coelacanth (Nikaido et al., 2013) and a grapevine genome (Patel et al., 2015). We applied a hybrid genome-assembly approach that uses short-read sequence and three mate-pair libraries, in combination with long-read sequences obtained from PacBio to improve sequence quality. The assembly performance was certified with the number of contigs, N_50_ length, repeat elements, BUSCO scores (Simão et al., 2015), distance between mapped BAC end sequences and whole-genome synteny to three reference citrus genomes by constructing pseudomolecules. Functional annotation verified the exactness of the predicted genes to the known gene families.

## Materials and methods

### Plant materials and genomic DNA isolation

All citrus plant materials used in this study were from the collection of the Division of Citrus Research, Institute of Fruit Tree and Tea Science. We selected Satsuma (*C. unshiu* Marc. strain “Miyagawa wase,” accession ID 117351), Kishu (*C. kinokuni* hort. ex Tanaka, strain “Mukaku Kishu,” accession ID 117399), kunenbo-A (*C. nobilis* Lour. var. kunep Tanaka, accession ID 117387), Clementine (*C. clementina* hort. ex Tanaka, accession ID 113161), sweet orange [*C. sinensis* (L.) Osbeck, strain “Trovita,” accession ID 172154], Willowleaf mandarin (*C. deliciosa* Ten., accession ID 117941), and pummelo “Banpeiyu” (*C. maxima* Merr., accession ID 171506). Their parentage was confirmed in our recent study (Shimizu et al., 2016b). The DNA samples from these trees were prepared for NGS analysis with a modified protocol using Nucleon Phytopure DNA extraction kit (GE Healthcare, Buckinghamshire, UK), and quality checked according to Shimizu et al. (2016a).

### Whole-genome sequencing

Both the paired-end tag and mate-paired-end tag were designed for whole-genome shotgun sequencing using the Illumina HiSeq 2000 platform (Illumina, CA, USA). The paired-end library was prepared by Illumina's TruSeq DNA PCR-Free Library Preparation Kit, according to the standard protocol. The read length was 101 bp. The paired-end tag indicated 300 bp genomic DNA fragments represented by a 320 bp median. The mate-paired libraries for 3, 5, and 8 kbp, were prepared with Illumina's TruSeq Mate Pair Sample Prep Kit, then provided for the paired-end sequencing. These short-read sequences were quality filtered to remove pairs that contained low-quality bases (QV < 10) at a proportion of 20% or higher, then contaminated reads were eliminated by mapping to the chloroplast genome of sweet orange (Bausher et al., 2006) and PhiX174 (NC_001422.1) with bwa (ver. 0.7.13) (Li and Durbin, 2009). The sequencing error was corrected with Musket (ver. 1.0.6) (Liu et al., 2013) to improve mapping quality at the default settings; next, all duplicated reads were excluded to retain unique sequencing reads. Finally, adapter sequences were trimmed using Trimmomatic (ver. 0.30) (Bolger et al., 2014). The numbers of the raw reads and the passed reads after the filtering process are summarized in Table S1.

Long-read sequence analysis was conducted with the PacBio RS II sequencer (Pacific Biosciences, CA, USA). A library for long-read sequencing was prepared with DNA Template Prep Kit 2.0 (3–10 kb). SMRT Cell Pac V3 and DNA Sequencing Kit 2.0 (PacBio) were used for the long-read sequencing. The obtained reads that passed with high QV scores (>20 for paired-end reads, >15 for PacBio reads) were used for the assembly (Table S1).

### Genome assembly, scaffolding, and finishing

We compared genome assemblers, SOAPdenovo2 (Luo et al., 2012), Velvet (Zerbino and Birney, 2008) and PLATANUS (Kajitani et al., 2014), for their performance to assemble the paired-end reads and mate-pair libraries, before the initial assembly. The number of produced scaffolds longer than 1,000 bp was 29,301; 38,998; and 12,247; the length of the longest scaffolds was 1.31, 3.00, and 4.00 Mb; and the N_50_ length of scaffolds was 52.8, 242, and 323 kbp, respectively. Consequently, we selected PLATANUS for the initial assembly. Genome assembly was conducted using the quality-filtered paired-end reads (Table S1) with PLATANUS (ver. 1.2.2) (Kajitani et al., 2014) in contig assembly mode; 322 Mb of the initial contigs were obtained. Next, three libraries of mate-pair sequencing (3, 5, and 8 kb) were integrated for scaffolding using PLATANUS in scaffolding mode. These paired-end reads, and mate-pair libraries were used again to close the gap using PLATANUS in gap close mode. The obtained scaffolds were optimized using Opera (ver. 1.3.1) (Gao et al., 2011) to improve the assembly quality and produced a 340.0 Mbp assembled sequence. We further attempted to close the gap in the obtained assembly with SMRT library read of PacBio by PBJelly (ver. 14.1.14) (English et al., 2014), then sequencing errors in the closed regions were corrected by mapping paired-end reads using bwa. The assembled sequences longer than 500 bp were selected for the finished sequence.

### Evaluation of the assembled sequence

The genome size of four citrus genomes (Satsuma “Miyagawa wase,” Clementine, pummelo “Banpeiyu,” and sweet orange “Trovita”) was estimated by *k-mer* analysis with quality-filtered reads of HiSeq 2000 by GenomeScope (Vurture et al., 2017) at *k* = 21. The quality of the assembled sequence and all predicted protein sequences were evaluated by BUSCO notation scores (Simão et al., 2015) and compared with other reference genome sequences. They were also evaluated for their assembly consistency by estimating a distance between the mapped position of both end sequences from same BAC clone of Clementine (Terol et al., 2008) to the same scaffold using BLASTN search for the genome sequences of haploid Clementine, diploid sweet orange (Wu et al., 2014), pummelo, doubled haploid sweet orange (ver.2), citron, papeda, and Atalantia (Wang et al., 2017) (reference genomes; RGs, hereafter). Statistical significance of these distances was evaluated by the Brunner-Munzel test in the lawstat package (Hui et al., 2008) of R (version 3.4.1, https://www.r-project.org), after eliminating any distance shorter than 1,000 bp or longer than 500 kb as outliers.

### Pseudomolecule construction and synteny analysis

The assembled Satsuma scaffolds were anchored to the three genetic maps for populations of Satsuma offsprings (population 1: 161 SSR, 512 SNP, population 2: 349 SSR, 476 SNP, and population 3: 278 SSR markers) by using SSR (Shimizu et al., 2016b) and SNP markers (Shimizu et al., 2016a; Minamikawa et al., 2017). Each genetic map consisted of 9 linkage groups of total lengths of 957, 1,017 and 919 cM, respectively. The anchored scaffolds were ordered and oriented according to the mapped position of the DNA markers on the genetic maps using ALLMAPS (Tang et al., 2015), then obtained 9 pseudomolecules. Whole genome synteny of the pseudomolecule to Clementine (Wu et al., 2014), pummelo and sweet orange (Wang et al., 2017) were visualized by the BLASTP search for their primary protein sequences with MCScanX (Wang et al., 2012).

### Repetitive sequence analysis

Repetitive sequences of the assembled Satsuma scaffold and RGs were initially modeled with RepeatModeler 1.0.8 (Smit and Hubley, 2016) using NCBI rmblast (ver. 2.2.28), RECON (ver.1.07), and RepeatScout (ver. 1.0.5). Modeled sequences longer than 500 nt were used for repetitive sequence identification with RepeatMasker (ver. open-4.0.6) (Smit et al., 2016). RepBase (ver. 20160829, http://www.girinst.org/) was used for the functional annotation of the identified sequence. Long terminal repeats (LTR) retrotransposons in the genome were mined with LTRharvest (Ellinghaus et al., 2008) without additional options, then annotated with LTRdigest (Steinbiss et al., 2009). Simple sequence repeat (SSR) regions in the assembled sequence were mined for motif lengths from 2 to 20 nt with mreps (Kolpakov et al., 2003).

### Gene prediction and functional analysis

Gene prediction of the assembled sequence was performed by gene modeling with MAKER-P (Campbell et al., 2014) pipeline. We first ran MAKER-P at the primary transcriptome detection mode (alternative splicing detection off), then obtained 24,168 genes and 24,207 mRNA. Another run in the “all transcriptome detection mode” (alternative splicing detection on) predicted 24,021 genes and 32,246 mRNA. The predicted gene and mRNA models obtained from these two different detection modes agreed each other, whereas approximately one-third did not. Thus, all of the genes and mRNA from these two different MAKER-P prediction models were merged, after which, we selected a unique set by eliminating redundant genes or mRNAs predicted at the same genome location. This procedure allowed us to obtain 29,024 predicted protein-coding genes and 37,970 mRNAs (8,946 splice variants).

The primary transcript sequences of RGs were used for ortholog search. Transcript sequences and their translated sequences of Arabidopsis were obtained at the *Arabidopsis* information portal (Araport11, https://www.araport.org/). Gene orthology of the predicted genes to the primary transcripts of RGs and *Arabidopsis* (Araport11) was deduced by the BLASTN or BLASTP programs.

Functional annotations of the predicted genes were performed in several ways. We evaluated the nucleotide sequences of the predicted genes with InterProScan (Jones et al., 2014; Finn et al., 2016) and performed similarity-based annotation analysis to the curated gene set of the model plant (Araport11) and UniProt (Bairoch et al., 2005; Wu et al., 2006). Functional annotations of the predicted genes were estimated by referring to the best hit of the InterProScan search at default settings and the curated gene ontology of the best-hit *Arabidopsis* gene in the Araport11 database by TBLASTX at the maximum threshold of E-value ≤ 1e-20. Metabolic pathway and orthology-oriented functional annotation of the nucleotide sequences of the predicted genes based on the KEGG database (Kanehisa et al., 2017) were estimated by KAAS (KEGG Automatic Annotation Server) (Moriya et al., 2007), which selected all dicot and monocot plants, all algae, and budding yeast, for reference organisms. The assigned annotations were sorted according to the KEGG BRITE functional hierarchy or by KEGG modules.

A phylogenetic evaluation of gene family was conducted by aligning the translated sequence with MUSCLE (Edgar, 2004) to obtain a distance matrix table. The evolutionary distances were computed using the Poisson correction method (Zuckerkandl and Pauling, 1965). The table was provided to develop a dendrogram by the Neighbor-Joining method (Saitou and Nei, 1987) at 1,000 bootstrap trails (Felsenstein, 1985). These analyses were conducted with MEGA7 (Kumar et al., 2016).

### Genome-wide allelic inconsistency analysis within trio genotypes

Paired-end short reads of parental varieties of Satsuma (Kishu “Mukaku Kishu” and kunenbo-A) (Shimizu et al., 2016b) and Clementine with its parental varieties (sweet orange “Trovita” and Willowleaf mandarin (Wu et al., 2014), were produced with Illumina's HiSeq 2000. These reads were mapped to the assembled sequence of Satsuma (this study) or the reference sequence of Clementine (Wu et al., 2014) by bwa (ver 0.7.13) (Li and Durbin, 2009), after quality filtering. The polymorphic loci among these two trios were detected and called variant sites of SNPs and short indels by SAMtools (ver 0.1.18) (Li et al., 2009). After excluding all indels or multiallelic SNP site, quality validated SNP sites in all trio varieties were used in the following analysis. Originally developed Perl programs computed the allelic inconsistency analysis of the two trio pedigrees for the selected SNPs.

## Results

### Genome sequencing and assembly

This study aimed to achieve the *de novo* assembly of the heterozygous diploid genome of Satsuma with sufficient quality for gene identification and genome-wide genotyping analysis. Different types of raw sequence reads, including short-read sequences with sufficient coverage, three mate-pair libraries for different insert lengths and long-read sequences of PacBio, were produced for this purpose (Table S1). According to the preliminary evaluation of three genome assemblers for their performance in assembling heterozygous Satsuma genome, we selected the PLATANUS (Kajitani et al., 2014) genome assembler, which was used for assembling the heterozygous genome in this study. After optimizing the scaffolds with three mate-pair libraries by Opera (Gao et al., 2011), raw assembled sequences with a total length of 348,354,554 bp, consisting of 20,973 scaffolds, were produced. The N_50_ length of the scaffolds was 386,934 bp before size elimination, but 12.5% of the nucleotides remained indeterminate (N ratio, hereafter). Finishing and gap closing was performed by mapping the long-read sequence of the PacBio to the raw assembled sequence with the PBJelly pipeline (English et al., 2014). The average read length of the PacBio reads was 2,418 bp for subreads and 1,992 bp for circular consensus sequence (CCS) reads (Table S1). Although initial mapping of the long reads with PBJelly slightly extended the total bases (359,893,593 bp) with no obvious changes for other scores (21,104 scaffolds and N_50_ length of scaffolds was 384,607 bp), it improved the confidence of sequences (7.9% of N ratio). Consecutive trials with PBJelly slightly improved the confidence (7 and 6.7% of N ratios at the second and the third run, respectively), but the N_50_ length of the scaffolds decreased to 382,542 bp and 379,361 bp, respectively. These degradations suggested the occurrence of fragmentation of the assembled sequence by over-fitting to the long-read sequences. Consequently, we selected the first assembled sequence, finished with long-read sequences by PBJelly.

As a result, an assembled sequence of the total length of 359,652,061 bp, consisting of 20,876 scaffolds was produced, after eliminating short sequences less than 500 bp or sequences that contained too many indeterminate nucleotides (Table 1). The total size of the assembled sequence was longer than those of the haploid Clementine (301.4 Mb), diploid sweet orange (319.2 Mb) (Wu et al., 2014), pummelo (345.8 Mb), and doubled haploid sweet orange (327.9 Mb) (Wang et al., 2017). The total size agreed with the expected genome size of the citrus and Satsuma (Ollitrault et al., 1994) and suggested the applicability of the PLATANUS assembler, combined with mapping of the long-read sequence. The composition of indeterminate nucleotides (7.84%) was higher than those of the haploid Clementine (2.06%) and pummelo (0.26%), but it was lower than those of the diploid sweet orange (20.9%) and the doubled haploid sweet orange sequences (8.16%). The number of the gaps in the sequence (35,790) was higher than that of the haploid Clementine (7,294) and pummelo (4,184), but close to that of the diploid sweet orange sequence (41,958) and the doubled haploid sweet orange (22,552). Thus, the hybrid assembly approach used in this study was deemed effective in reducing ambiguous nucleotides of the heterozygous diploid genome by integration with long-read sequences.

**Table 1:** Summary of the draft sequence of the Satsuma genome.

**Features**	**Numbers**
Assembly total size	359,652,061 bp
Total number of scaffolds	20,876
Longest scaffold	5,227,725 bp
N_50_ size of scaffold	386,404 bp
G-C % ratio	33.9 %
Gaps	35,790
N ratio	7.84 %
Repeat masked	39.52 %
LTR retrotransposon	7,950
Simple sequence repeat (SSR,2–20 bp motif)	203,795
Genes	29,024
mRNA	38,838

### Evaluation of the assembled scaffold by BUSCO and BAC end sequence mapping analysis

The estimated genome size of Satsuma, Clementine, pummel, and sweet orange by *k-mer* analysis with Genome Scope (Vurture et al., 2017) were 278.0, 274.0, 283.6, and 269.8 Mb, respectively. These estimated sizes were apparently smaller than the obtained genome size of Satsuma (359.7 Mb, Table 1) and previously reported sizes of Clementine (301.4 Mb) (Wu et al., 2014), pummelo (345.8 Mb), and sweet orange (327.9 Mb) (Wang et al., 2017). The estimated genome sizes by *k-mer* analysis were also lower than those reported by Ollitrault et al. (373, 400.3, and 387.0 Mb for Satsuma, pummelo and sweet orange, respectively) (Ollitrault et al., 1994) or by Loureiro et al. (425 Mb for sweet orange) (Loureiro et al., 2007). Another *k-mer* analysis by a different software yielded low value, too (data not shown). The heterozygosity (*H*o) of Satsuma, Clementine, pummelo and sweet orange were 0.435, 0.462, 0.022, and 0.716 (Shimizu et al., 2016a), respectively. No direct relationship between the heterozygosity and the estimated size was suggested.

The BUSCO score for the assembled scaffolds was 94.2% (Table 2). It was close to that of Clementine and higher than that of diploid sweet orange (Table 2). Other BUSCO scores were also higher than diploid sweet orange or comparable to the genome sequences of haploid or doubled haploid (Table 2). The N_50_ length of the distance between both end sequences from the same BAC clone that was mapped to the same scaffolds or chromosomes showed similar distances for Satsuma and other seven citrus genomes and indicated the consistency of genome assembly (Table 3). These scores demonstrated that the method used in this study produced a sequence from the diploid genome of a quality comparable to that obtained from the haploid genome.

**Table 2:** BUSCO notation scores of the Satsuma and four reference citrus genomes.

**Genome assembly**	**Size**	**BUSCO notation assessment scores**
Satsuma^1^	20,876 scaffold	C:94.2% [S:92.3%, D:1.9%], F:2.2%, M:3.6%, n:1440
	37,970 peptides	C:92.1% [S:64.2%, D:27.9%], F:4.4%, M:3.5%, n:1440
Clementine^2^	1,398 scaffolds	C:94.3% [S:92.1%, D:2.2%], F:2.0%, M:3.7%, n:1440
	33,929 peptides	C:94.8% [S:71.5%, D:23.3%], F:2.4%, M:2.8%, n:1440
Sweet orange diploid (ver 1.1)^2^	12,574 scaffolds	C:89.7% [S:87.6%, D:2.1%], F:3.6%, M:6.7%, n:1440
	46,147 peptides	C:87.5% [S:52.8%, D:34.7%], F:6.3%, M:6.2%, n:1440
Pummelo^3^	Pseudomolecule	C:95.6% [S:92.8%, D:2.8%], F:1.4%, M:3.0%, n:1440
	42,886 peptides	C:94.5% [S:65.6%, D:28.9%], F:2.6%, M:2.9%, n:1440
Sweet orange DH (ver 2)^3^	Pseudomolecule	C:95.3% [S:92.6%, D:2.7%], F:1.7%, M:3.0%, n:1440
	44,275 peptides	C:94.4% [S:60.2%, D:34.2%], F:2.3%, M:3.3%, n:1440

*C, complete; S,single-copy; D,duplicated; F,fragmented; M,missing; n, gene number*.

1) *This study*,

2) *Wu et al. (2014) Nat. Biotechnol. 32, 656-662*,

3) *Wang et al. (2017) Nat. Genet. 49, 765-772*.

**Table 3:** Evaluation of the assembled genome consistency by BAC end sequence mapping analysis.

**Genome assembly**	**Type of sequence**	***N***	***N*_50_**	**Mean**	***SD***	**Sig**
Satsuma^1^	Scaffolds	8,202	112,797	115,162	36944.8	a
Satsuma^1^	Pseudomolecule	7,658	113,317	118,361	56002.5	ab
Clementine^2^	Scaffolds	16,914	117,789	121,056	40684.1	c
Pummelo^3^	Pseudomolecule	11,222	116,172	123,387	57490.2	d
SO DHv2^3^	Pseudomolecule	12,353	124,168	128,875	46844.3	e
SO diploid^2^	Scaffolds	6,545	112,739	113,832	35403.1	abf
Citron^3^	Scaffolds	4,011	111,039	115,588	49750.2	abfg
Papeda^3^	Scaffolds	6,811	116,602	118,191	49404.1	dh
Atalantia^3^	Scaffolds	6,482	123,148	126,367	52667.8	i

*N, Number of Clementine BAC clones that were mapped with both end sequences to the same scaffold/pseudomolecule*.

*N_50_, mean, median and average of the distance (bp) between both end sequences of a Clementine BAC clone that were mapped to the same scaffold/pseudomolecule*.

*Sig, different letters represent significance between them at p < 0.05 by the Brunner-Munzel test*.

1–3: *see the legend of Table 2*.

### Pseudomolecule construction and synteny analysis

**Figure 1:**
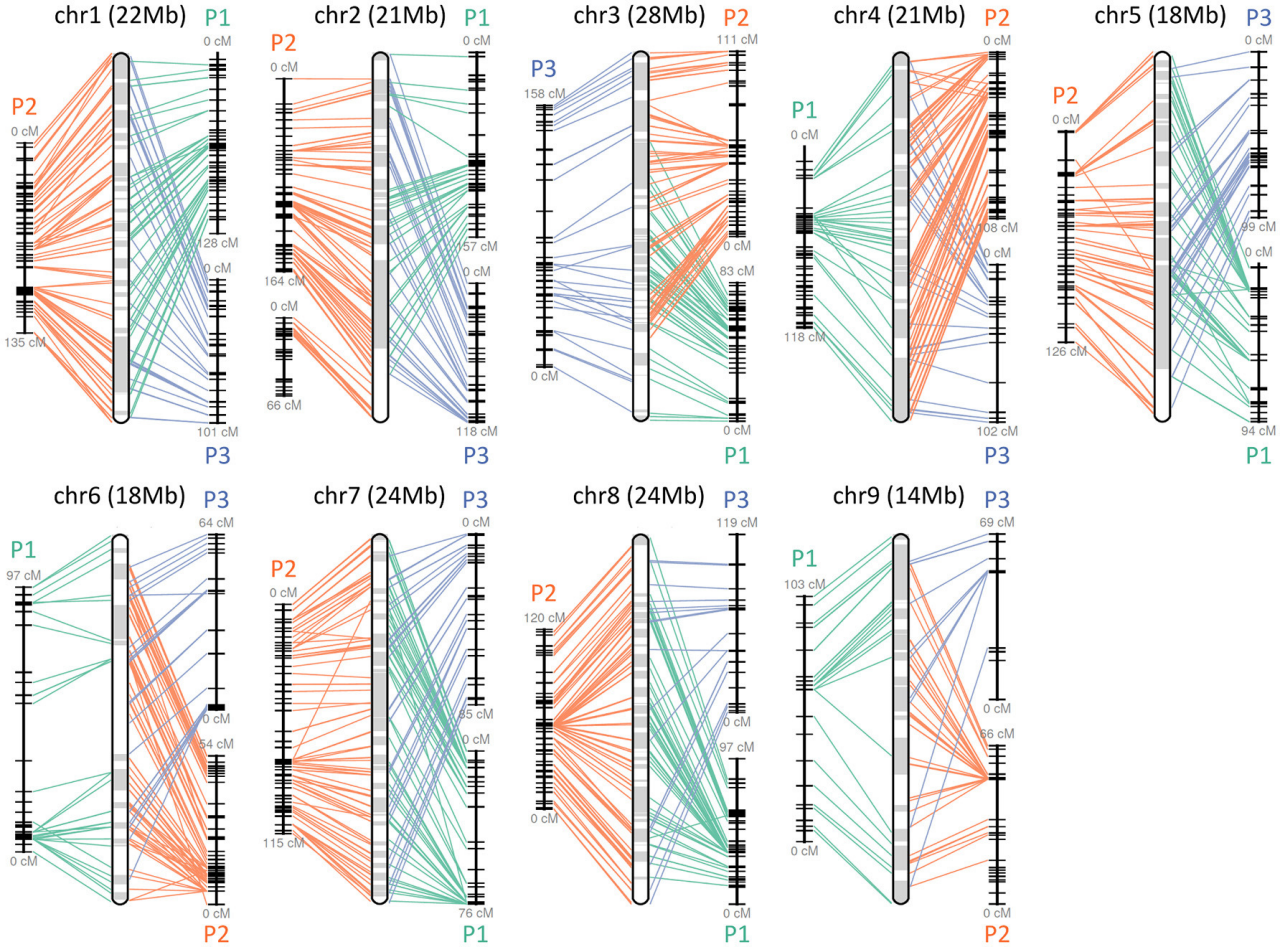
Pseudomolecule construction of Satsuma by aligning the scaffolds to the three genetic maps. Chr 1 to 9 represents constructed pseudomolecules by merging three genetic maps of Satsuma offsprings. Numbers in parenthesis indicate the length of constructed pseudomolecule. Central round rectangle is a schematic diagram of the merged pseudomolecule. P1 (green), P2 (orange), and P3 (blue) of each side correspond to the genetic maps of population 1 (161 SSR, 512 SNP, 957 cM), population 2 (349 SSR, 476 SNP, 1,017 cM), and population 3 (278 SSR, 919 cM), respectively. Each line denotes a DNA marker that was mapped to the scaffold and applied for scaffold assembly.

**Figure 2:**
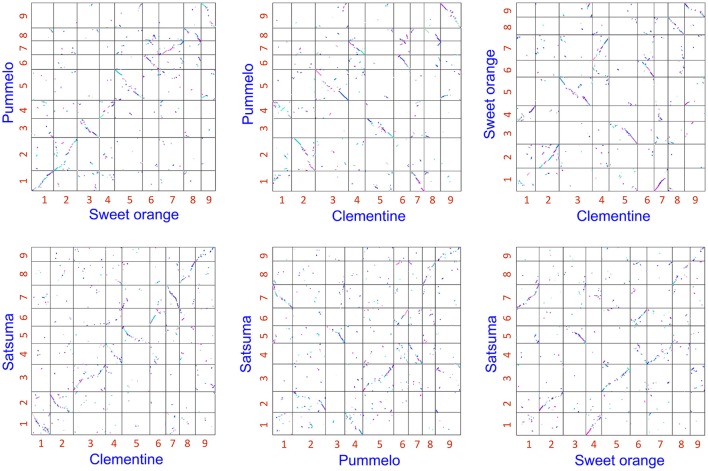
2-D genome synteny plot among Satsuma pseudomolecule and three reference citrus genomes.Satsuma: 9 pseudomolecules (this study), Clementine: scaffold 1 to 9 from Wu et al. (2014). Pummelo and sweet orange: chromosome 1 to 9 from Wang et al. (2017). Numbers on each axis correspond to their numbers of chromosome or scaffold.

The obtained Satsuma scaffolds were further merged to construct pseudomolecules by aligning them to three genetic maps with SSR and SNP markers, then 9 pseudomolecules with a total length of 189.6 Mbp were obtained (Figure 1, Table 4). The total length of the pseudomolecules was 52.7% of the total length of the Satsuma scaffolds, but N ratio showed a slight improvement from 7.84% (Table 1) to 5.02% (Table 4). Whole genome alignment analysis of the pseudomolecules (Figure 2) revealed one-to-one coincidence to those of 9 scaffolds of Clementine (Wu et al., 2014) and 9 chromosomes of pummelo and sweet orange genomes (Wang et al., 2017). Their lengths ranged from 35.5 to 112.5% of the corresponding scaffolds or chromosomes in other citrus genomes (Table 4). The observed coincidence among those citrus genomes confirmed their synteny in the citrus genome and suggested availability for further genome-wide analysis of the Satsuma genome.

**Table 4:** Size ratio of Satsuma pseudomolecule to the corresponding scaffold or chromosome of the reference citrus genomes.

**Chr**	**Satsuma pseudomolecule**^1^		**Size ratio to the reference citrus genomes**					
	**Size**	***N* ratio (%)**	**Clementine**^2^		**Pummelo**^3^		**Sweet orange DH ver2**^3^	
1	22,490,505	4.56	77.7%	(scaffold_1)	76.6%	(chr4)	112.7%	(chr4)
2	21,324,674	2.99	58.6%	(scaffold_2)	40.2%	(chr2)	69.2%	(chr2)
3	27,672,184	5.54	54.2%	(scaffold_3)	55.9%	(chr5)	76.6%	(chr5)
4	20,933,864	3.31	81.6%	(scaffold_4)	94.0%	(chr7)	65.0%	(chr7)
5	17,746,050	4.94	41.0%	(scaffold_5)	57.9%	(chr3)	61.8%	(chr3)
6	17,666,241	5.43	69.0%	(scaffold_6)	74.7%	(chr6)	83.4%	(chr6)
7	23,734,688	6.36	112.3%	(scaffold_7)	74.0%	(chr1)	82.4%	(chr1)
8	23,655,052	6.73	94.2%	(scaffold_8)	112.5%	(chr8)	104.2%	(chr8)
9	14,331,940	4.82	45.6%	(scaffold_9)	35.5%	(chr9)	77.7%	(chr9)
Sum	189,555,198	5.02	65.7%		62.8%		79.3%	

1–3 *see the legend of Table 2*.

### Repeat element analysis

Repeat modeling of the assembled sequence with RepeatModeler estimated 926 repeat sequences (34–10,481 bp, average 1,448 bp). Repeat sequences predicted by RepeatMasker with the repeat model sequences longer than 500 bp (696 sequences, average 1,839 bp) identified repeat elements that consisted of 39.52% of the assembled sequences (Table 5, Table S2). The long interspersed nuclear elements (LINE) accounted for 1.44% of the sequence, but no short interspersed nuclear elements (SINE) was identified (Table S2). Retrotransposable elements with LTR sequences predicted by RepeatMasker were the primary class of repeat sequences that occupied 21.58% of the genome and Ty3-*Gypsy* or Ty1-*Copia* type retrotransposable elements were the dominant types (Table S3).

**Table 5:** Summary of the detected repeat elements of Satsuma and seven reference citrus genome sequences.

**Genome assembly**	**Satsuma^1^**	**Clementine^2^**	**Pummelo^3^**	**Sweet orange DH ver2^3^**	**Sweet orange diploid^2^**	**Citron^3^**	**Papeda^3^**	**Atalantia^3^**
**(A) DEDUCED REPEAT ELEMENTS IN THE GENOME SEQUENCES**								
Sequences	20,876	1,398	10	10	12,574	32,732	14,916	25,600
Total length (bp)	359,652,061	301,386,998	345,779,982	327,944,670	319,231,331	406,057,947	357,621,246	315,820,821
GC%	33.9	35.0	35.0	34.1	34.6	35.2	34.2	33.6
Bases masked (bp)	142,143,773	140,273,551	173,037,803	133,788,768	105,017,054	177,784,835	146,385,102	119,147,866
(masked %)	39.5	46.5	50.0	40.8	32.9	43.8	40.9	37.7
Sequences with repeats	18,990	1,390	10	10	12,272	27,105	11,963	18,565
(%)	91.0%	99.4%	100.0%	100.0%	97.6%	82.8%	80.2%	72.5%
**(B) BRIEF CATEGORIES OF THE DEDUCED REPEATS IN THE GENOME SEQUENCES**								
SINE	0.00	0.02	0.11	0.01	0.00	0.08	0.01	0.02
LINEs	1.44	1.69	1.96	1.64	1.70	1.77	1.75	1.26
LTR elements	21.6	19.9	25.5	18.8	18.1	22.9	18.8	17.6
DNA elements	5.15	6.11	5.42	5.92	3.81	4.65	6.08	5.60
Unclassified	9.8	14.6	12.6	12.3	8.0	8.9	12.0	11.4
Satellites	0.02	0.04	0.00	0.05	0.00	0.00	0.04	0.02
Simple repeats	1.44	3.94	2.47	1.81	1.09	5.15	2.09	1.56
Low complexity	0.28	0.27	0.27	0.28	0.23	0.26	0.29	0.29

*Percent ratios of the deduced repeats to the total genome length*.

1–3: *see the legend of Table 2*.

Several types of DNA transposable elements occupied considerable portions of the genome (5.15%, Table S2), and hAT-Ac type transposon was dominant among the DNA elements (Table S3). Simple repeats that included SSRs were found in 1.44% of the genome (Table S2). These estimated repeat elements and the ratios to the assembled genome were similar to those reported in the haploid Clementine (Wu et al., 2014); comprehensive analysis of the repeat elements of the RGs by the same manner showed similar scores (Table 5).

Further evaluation with LTRharvest (Ellinghaus et al., 2008) and LTRdigest (Steinbiss et al., 2009) detected 7,950 LTR retrotransposons that accounted for 13.2% of the assembled genome (Table 6A). The compositions of LTR retrotransposons in the other six citrus genomes detected by the same tools were comparable, except for the diploid sweet orange genome, which exhibited fewer elements (Table 6A). Functional annotation of the detected elements predicted 310 LTR retrotransposons contained in all components (primer binding site; PBS, and *gag* and *pol* genes) with average LTR similarity of 93.6% (max 100%) which were presumed to retain activity for transposition (Table 6B). The mining of SSRs identified 203,795 regions in the assembled sequence from 2 to 20 nt motif size (Figure S1). The number of the identified SSRs was comparable to that of the haploid Clementine genome (319,630), pummelo (373,547), and the doubled haploid sweet orange genome (334,552) which were estimated in the same manner, but fewer were identified in the diploid sweet orange genome (252,364).

**Table 6:** Summary of the retrotransposon of Satsuma and seven reference citrus genomes.

**Genome assembly**	**Satsuma^1^**	**Clementine^2^**	**Pummelo^3^**	**Sweet orange DH ver2^3^**	**Sweet orange diploid^2^**	**Citron^3^**	**Papeda^3^**	**Atalantia^3^**
**(A) SUMMARY OF THE DEDUCED RETROTRANSPOSON**								
Deduced retrotransposons	7,950	7,300	7,580	8,737	4,096	9,519	9,529	6,793
Minimum size (bp)	1,112	1,110	1,128	1,131	1,130	1,122	1,127	1,145
Maximum size (bp)	15,979	15,980	15,998	15,973	15,954	15,995	15,931	15,966
Avg size	5,969.4	6,426.7	7,046.0	5,683.3	6,522	6,204	5,867.6	5,680.2
Total length	47,456,750	46,914,849	53,408,728	49,654,779	26,712,742	59,053,325	55,912,382	38,585,423
(% genome)	13.20%	15.57%	15.45%	15.14%	8.37%	14.54%	15.63%	12.22%
Overlapped to the	3,066							
deduced genes	(38.6%)							
Genome size (bp)	359,652,061	301,386,998	345,779,982	327,944,670	319,231,331	406,057,947	357,621,246	315,820,821
**(B) DETAIL SUMMARY OF THE DEDUCED RETROTRANSPOSON ACCORDING TO THEIR FUNCTIONAL ELEMENT AND GENES**								
+PBS	4,020							
+gag	1,242							
+pol	2,688							
+PBS+gag+pol	310							
Average LTR similarity: 93.6%								
(Max 100–min 85.4%)								

*PBS: primer binding site, gag: gene encodes the virus-like particle (VLP), and pol: gene encodes a reverse transcriptase (RT) and related proteases*.

1–3: *see the legend of Table 2*.

### Gene prediction with Maker-P and orthology

Gene prediction by gene modeling with the MAKER-P annotated 29,024 predicted protein-coding genes (PCGs) in 2,540 scaffolds, for an average total length of 3,224 bp and average coding sequence length of 1,167 bp (Table 7). The numbers of PCGs were in agreement with those reported for the RGs. Among the PCGs, 5,676 genes (19.6%) overlapped other genes because of two separate runs of MAKER-P with different prediction models. Although 4,945 PCGs overlapped with another gene, 731 PCGs overlapped to two to six other genes. The average number of exons per gene was 4.11, with an average exon size of 287.0 bp (Table 7).

**Table 7:** Gene modeling and protein-coding gene prediction by MAKER-P.

**Features of the predicted genes**	**Numbers**	
Predicted genes	29,024	
Mean gene length (bp)	3,224	
Longest gene length (bp)	47,871	
Shortest gene length (bp)	129	
Genes with no intron	4,634	(16.0%)
Genes overlapped	5,676	(19.6%)
**Features of the predicted transcripts**	**Primary transcript**	**All transcript**
No. of mRNAs	29,024	37,970
Mean transcript length (bp)	1257.5	1604.5
Mean CDS length (bp)	1166.7	1256.3
Mean protein length (aa)	387.9	514.8
Mean exon number	4.11	3.15
Mean exon length (bp)	287.0	239.9

**Figure 3:**
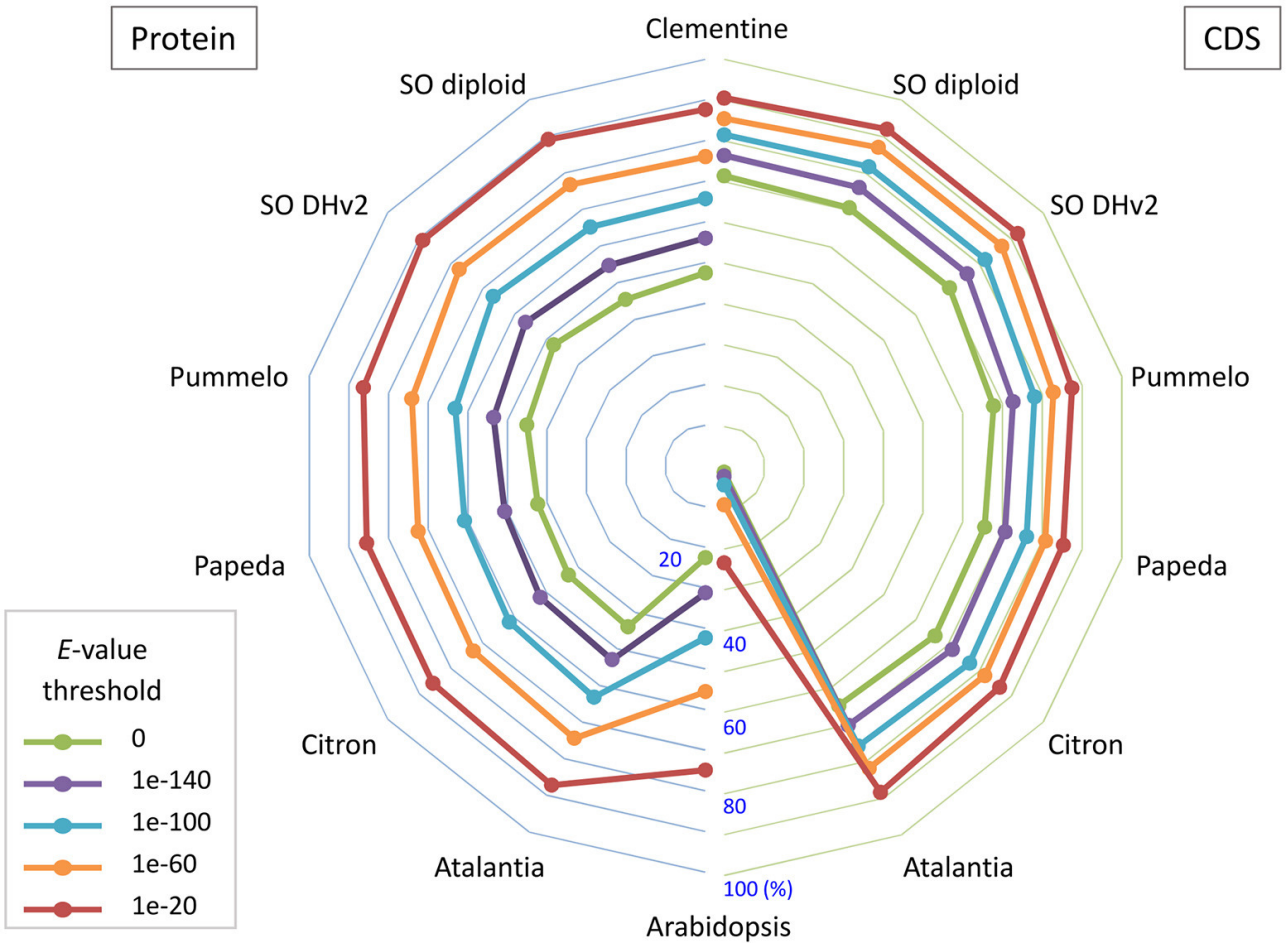
The bipartite spider plot of the ratio of orthologous gene of Satsuma to the seven citrus genomes and *Arabidopsis* for their protein coding sequence and translated sequence. Individual symbols indicate the ratio of primary genes that were orthologs to seven reference genomes for their nucleotide sequence (CDS; right) by BLASTN or protein sequence (Protein; left) by BLASTP. Half circle lines on the left and right sides represent the ratio at the central (%). Each line shows the ratio at threshold *E*-value for the homology evaluation.

Primary transcripts of Satsuma showed higher homologies to Clementine and sweet orange than pummelo, papeda, citron, and Atalantia by BLASTN search for protein coding sequence (CDS) and BLASTP search for protein sequence at five threshold levels, as expected from their proposed phylogeny (Figure 3). Over 71% of the PCGs were supported by at least one highly significant BLASTN hit (*E*-value ≤ 1 × 10^−100^) for the transcripts of RGs. Among them, 1,761 of the primary transcripts showed no significant homology to those of RGs, even at 1E-40 threshold, but 495 of them were annotated from Arabidopsis orthologs or InterProScan. Among the primary CDS, 1,587 (5.47%) lacked the ATG start codon, 1,550 (5.34%) did not have any of the three stop codons (UGA, UAA, or UAG) and these CDS lacked a start codon as well. Most used stop codon was UGA (11,552), followed by UAA (9,510) and UAG (6,412). The ratio of the SSR found in the predicted exon region to the total number of SSRs at each motif length showed periodical oscillation at 3, 6, 9, 12, and 15 nt motif lengths, but no similar oscillation was observed in the untranscribed region (UTR) of the assembled sequence (Figure S1). These periodical oscillation implied selection within the coding region other than the UTR region to maintain proper functionality of the translated product. Similar motif size-dependent differences in the occurrence of SSR sequences between UTR and ESTs have been reported (Gmitter et al., 2012). The BUSCO score of the predicted proteins was slightly lower than that of other citrus genomes, but higher than that of diploid sweet orange (Table 2). These observations suggested that the number and accuracy of the predicted transcript of Satsuma were higher than the previous diploid genome and comparable to those of the haploid genomes.

### Functional annotation and gene families of the predicted gene

#### Functional annotation and gene ontology (GO) of the predicted genes

A TBLASTX search of the translated sequences of PCGs to the curated cDNA of *Arabidopsis* annotated with gene ontology (GO), indicated significant similarities for the 22,427 genes (77.3%) with the threshold at *E*-value ≤1 × 10^−20^. Another similarity search by InterProScan at the default threshold indicated significant similarities with protein signatures to more than 80% of the PCGs and 22,351 (77.0%) were annotated. Consequently, more than 85% of the PCGs were annotated successfully by retrieving the curated annotation of *Arabidopsis* cDNA or InterProScan.

**Figure 4:**
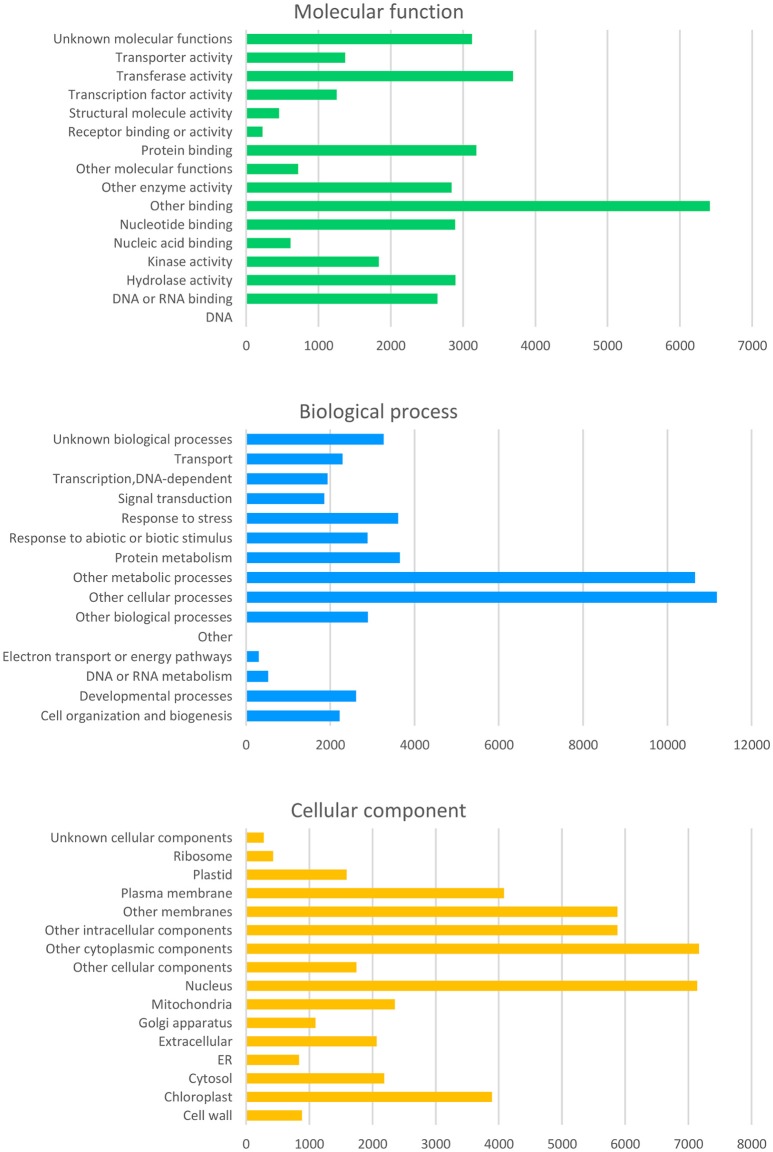
Distribution of the deduced gene ontology (GO) of the Satsuma primary genes. Each panel represents the number of GO slim annotations for molecular function, biological process, and cellular component for the predicted coding genes of the Satsuma mandarin. They were retrieved by similarity to the curated cDNA of *Arabidopsis* with the threshold of *E*-value ≤ 1 × 10^−20^.

From the curated GO annotations of *Arabidopsis* and InterProScan, a total of 141,493 GO annotations were retrieved for the predicted 22,308 genes from *Arabidopsis* and 45,499 GO annotations from InterProScan for the 11,936 PCGs. As a result, 23,891 genes (82.3%) held at least one GO annotation from *Arabidopsis* or InterProScan. Another similarity search of the 8,946 splice variant sequences (SV) retrieved GO of 8,009 (89.5%) and 6,219 (69.5%) with those of *Arabidopsis* and InterProScan, respectively. The number of the retrieved GO slim annotations in three categories (molecular function, biological process, and cellular component) showed a disproportional distribution (Figure 4) similar to that of other citrus genomes (data not shown).

#### Gene families

Functional annotations with the curated *Arabidopsis* database classified 8,040 PCGs into 60 repertoires of gene families (Table S4). Except for the 3,817 PCGs classified to the miscellaneous families (“NULL” in the Table S4), the “acyl lipid metabolism family” was the largest family, assigned with 562 PCGs in 163 gene families; while the “cytochrome P450 family” (341 PCGs) and the “glycosyltransferase gene family” (324 PCGs) followed (Table S4). The “ATP-binding cassette (ABC) transporters” is an important protein family involved in the transport of plant secondary metabolites (Yazaki, 2006; Kang et al., 2011) in which, 294 PCGs were classified. Likewise, at least seven transcriptional factor families were identified for 326 PCGs. One hundred twenty-eight of these were basic helix-loop-helix (bHLH) transcription factors, and 107 PCGs were classified as *myb* transcriptional factors (Table S4). These gene families predicted from orthologs of *Arabidopsis* agreed well with those of protein signatures inferred from InterProScan.

#### Feature analysis of the predicted genes

An automatic annotation of PCGs with KAAS (Moriya et al., 2007) classified 5,948; 3,723; and 1,027 PCGs into three KEGG BRITE hierarchies of protein families for metabolism, genetic information processing and signaling and cellular processes, respectively (Table S5). Among these, 5,948 PCGs were classified into 101 top classes in 11 protein families for metabolism, and 2,136 were mapped to KEGG pathways (Table S5). More than 4,500 PCGs were associated with a BRITE hierarchy “enzymes,” followed by “protein kinases,” “peptidases,” and “glycosyltransferases.” The 3,723 PCGs classified to “genetic information processing” families were associated with 35 top classes in 16 protein families and 345 were assigned to a transcription factor. The 1,027 PCGs classified to “signaling and cellular processes” families were associated with 20 top classes in 10 protein families, but most of them were related to “exosome” (Table S5). Another KAAS analysis classified 3,399 PCGs into four KEGG modules, pathways, structural complexes, functional sets, and signature modules; 3,358 PCGs were mapped to the KEGG pathways (Table S6).

#### Cytochrome P450 family genes

The P450 family (CYP) is one of the largest gene families in plant genomes (Bak et al., 2011; Nelson and Werck-Reichhart, 2011; Mizutani, 2012). It is a group of heme-thiolate enzymes that catalyze diverse and important secondary metabolic reactions by monooxygenase activity (Werck-Reichhart and Feyereisen, 2000; Mizutani, 2012); between 202 and 292 CYP genes have been reported in citrus genomes (Mittapelli et al., 2014). Because various CYP genes are recognized to be involved in broad, important physiological processes in plants (Vranová et al., 2012; Magome et al., 2013; Hedden and Sponsel, 2015; Yuan et al., 2015), sorting and classifying the CYP gene family is the first step toward understanding key reactions in these processes. The mining of CYP genes in Satsuma by using KAAS annotation or a BLAST search to determine CYP genes in *Arabidopsis* resulted in 260 candidate CYP genes in 46 clans (Table 8, Table S7). The dendrogram of the deduced P450 gene family classified each family member according to their major clans and verified this family (Figure S2). Although the number of the deduced CYP genes was slightly lower than that reported for the Clementine genome, the number of CYP genes in multiple family clans (CYP71 clan, CYP72 clan, CYP 85 clan, and CYP86 clan) was proportional to that reported in the three RGs (Mittapelli et al., 2014). These diverse CYP families were widely found in mosses, gymnosperms, and angiosperms (Nelson and Werck-Reichhart, 2011); they were considered to enhance secondary metabolism in plants (Mizutani, 2012). The number of CYP genes in single-family clans (CYP51, CYP74, CYP97, CYP710, and CYP711 clans) that have been conserved among algae, mosses, and higher plants were well-conserved among Satsuma and the three RGs (Mittapelli et al., 2014). These genes in single-family clans are believed to be involved in core metabolism in plants or phytohormone homeostasis (Mizutani, 2012) and these conserved CYP genes among citrus genomes coincided in their roles.

**Table 8:** Family wise distribution of cytochrome P450 genes in Satsuma.

**CYP clan**	**No. of families**	**No. of genes in *C. unshiu***
**A-TYPE CYTOCHROME P450**		
CYP71 clan	19	166
**Non-A-TYPE CYTOCHROME P450**		
CYP51 clan	1	1
CYP72 clan	8	23
CYP74 clan	1	3
CYP85 clan	10	32
CYP86 clan	4	25
CYP97 clan	1	7
CYP710 clan	1	1
CYP711 clan	1	2
Total	46	260

#### Genes responsible for isoprenoids and gibberellic acid biosynthesis

Isoprenoids (also called terpenoids) are the largest group of natural products synthesized from the five-carbon building unit isopentenyl diphosphate (IPP) (Dewick, 1997). More than 25,000 compounds have been identified for isoprenoids in living organisms (Croteau et al., 2000). Wide varieties of pigments, aromas, phytohormones, lipids and waxes are of isoprenoid origin (Dewick, 1997; Croteau et al., 2000; Fraser and Bramley, 2004; Hedden and Sponsel, 2015). These compounds have essential roles, including regulating growth, flowering, stress tolerance, fruit setting, and fruit quality (Fleet and Sun, 2005; Iglesias et al., 2007; Leng et al., 2014). In plants, two pathways for IPP biosynthesis, the mevalonic acid (MVA) pathway that starts from acetyl-CoA and the methylerythritol 4-phosphate (MEP) pathway that starts from pyruvate and glyceraldehyde 3-phosphate (GA-3P), supply IPP to the cytosol and plastids, respectively (Dewick, 1997; Croteau et al., 2000; Okada, 2011; Pulido et al., 2012; Vranová et al., 2012). These two pathways are not isolated from one another, but the MVA pathway is proposed to supply IPP or other intermediates to plastids in etiolated seedlings under dark conditions (Vranová et al., 2012). In Satsuma, gene mining identified the genes for each step in the MVA and MEP pathways (Table S8, Figure S3). The MEP pathway supplies IPP to the downstream pathway toward gibberellic acid and carotenoid biosynthesis in plastids. The gene mining identified at least one to three types of genes in each of seven steps in this pathway in Satsuma (Table S8, Figure S3). The geranylgeranyl diphosphate (GGPP) synthase (GGPS) catalyzes the production of GGPP under both pathways and this was comprised of 11 genes in *Arabidopsis* (Ruiz-Sola et al., 2016). Most of those GGPSs were located at cytosol, but only GGPS11 is an isozyme that is transported in plastids (Ruiz-Sola et al., 2016). In Satsuma, five genes for three isozymes (GGPS6, GGPS10 and GGPS11) were identified (Table S8).

**Figure 5:**
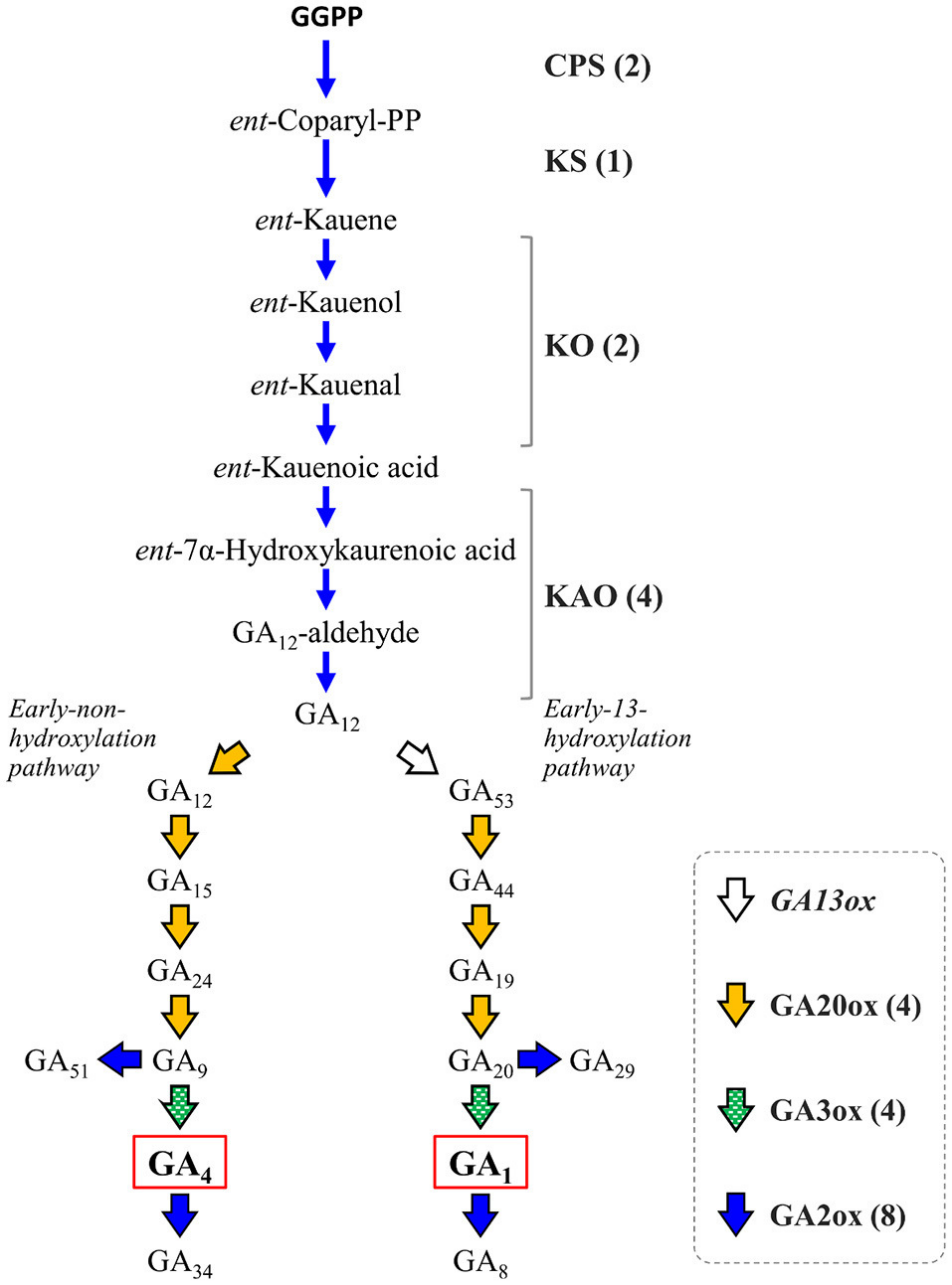
Genes involved in the biosynthesis and deactivation of bioactive gibberellic acid in Satsuma. GGPP, geranylgeranyl diphosphate; GAxx, gibberellic acid xx; CPS, *ent*-copalyl diphosphate synthase; KS, *ent*-kaurene synthase; KO, *ent*-kaurene oxidase; KAO, *ent*-kaurenoic acid oxidase; GA13ox, gibberellin 13-oxidase, putative; GA20ox, gibberellin 20-oxidase; GA2ox, gibberellin 2-oxidase; GA3ox, gibberellin 3-oxidase. Numbers in parentheses represent the number of each detected gene.

Gibberellins (GAs) are a group of endogenous plant growth regulators that promote plant growth and affect the morphology of various organs (Hedden and Kamiya, 1997; Fleet and Sun, 2005; Hedden and Sponsel, 2015). GAs are tetracyclic diterpenoid compounds synthesized from GGPP as the substrate through an eight-step reaction by the action of four enzymes: *ent*-copalyl diphosphate synthase (CPS), *ent*-kaurene synthase (KS), *ent*-kaurene oxidase (KO or CYP701A3), and *ent*-kaurenoic acid oxidase (KAO) whose activity yields a common precursor, gibberellic acid 12 (GA_12_). In citrus, GAs have been recognized to be involved in parthenocarpy (Talon et al., 1990, 1992), fruit setting (Ben-Cheikh et al., 1997), flower induction (Goldberg-Moeller et al., 2013) and coloring of fruits (Alós et al., 2006). Satsuma exhibits stronger parthenocarpy than other citrus varieties, but the molecular mechanism underlying induction of fruit set, as well as the regulation of molecular biosynthesis, are not fully elucidated yet. Mining the genes of Satsuma for these initial precursor synthesis steps from GGPP to GA_12_ identified one to four genes in each step (Figure 5, Table S8) and the number of these genes were consistent with those of the Clementine (data not shown). The bioactive gibberellins (GA_1_ or GA_4_ in citrus) are produced from GA_12_ in the early non-hydroxylation pathway or the early 13-hydroxylation pathway by at least two enzymes, gibberellin 20-oxidase (GA20ox) and gibberellin 3-oxidase (GA3ox). The GA20ox enzyme converts GA_12_ to GA_9_ or GA_20_ in a stepwise manner to supply the direct precursors of bioactive GAs in both pathways. Mining of Satsuma genes deduced four GA20ox genes, and two of them were matched to the two GA20ox genes that were recently revealed to be functional in Satsuma (Kotoda et al., 2015). The GA3ox enzyme converts the precursor GA_9_ or GA_20_ to bioactive GAs, then gibberellin 2-oxidase (GA2ox) degrades bioactive GAs and inactivates them. The actions of GA3ox or GA2ox are the key to regulating the content of biologically active GAs in plants (Hedden and Sponsel, 2015). Although no GA3ox genes have been characterized in citrus, gene mining deduced four GA3ox candidate genes in Satsuma (Figure 5, Table S8), which are the orthologs of GA3ox1 and GA3ox2 in *Arabidopsis*. Four orthologs of GA3ox were found in the genes of the Clementine genome (data not shown). The eight genes of Satsuma were deduced to be GA2ox and three of them matched to the functional GA2ox genes that were recently reported in Satsuma (Kotoda et al., 2017). They were the orthologs of GA2ox1, 2, 4, 6, and 8 of *Arabidopsis*. Although GA_1_ is a major active GA in citrus (Talon et al., 1992), the early 13-hydroxylation pathway in GA biosynthesis is not yet fully elucidated in citruses. Gibberellin 13-oxidase (GA13ox) is a cytochrome P450 family enzyme (CYP714B) and is known to catalyze the 13-hydroxylation process of GA compounds in rice (Magome et al., 2013). The CYP714B orthologs have not been identified in citrus or *Arabidopsis*; however, gene mining detected two CYP714A genes and one CYP714C gene in Satsuma (Table S8). Functional analysis of *Arabidopsis* CYP714A genes revealed they contribute to the production of diverse GA compounds and suggested a different function for them. These identified genes were consistent with the current understanding of their roles in these pathways. Thus, further analysis is anticipated to reveal the involvement of CYP714A or CYP714C orthologs in the 13-hydroxylation process in citrus.

#### Genes involved in the biosynthesis of carotenoids to abscisic acid and the light acclimation system

**Figure 6:**
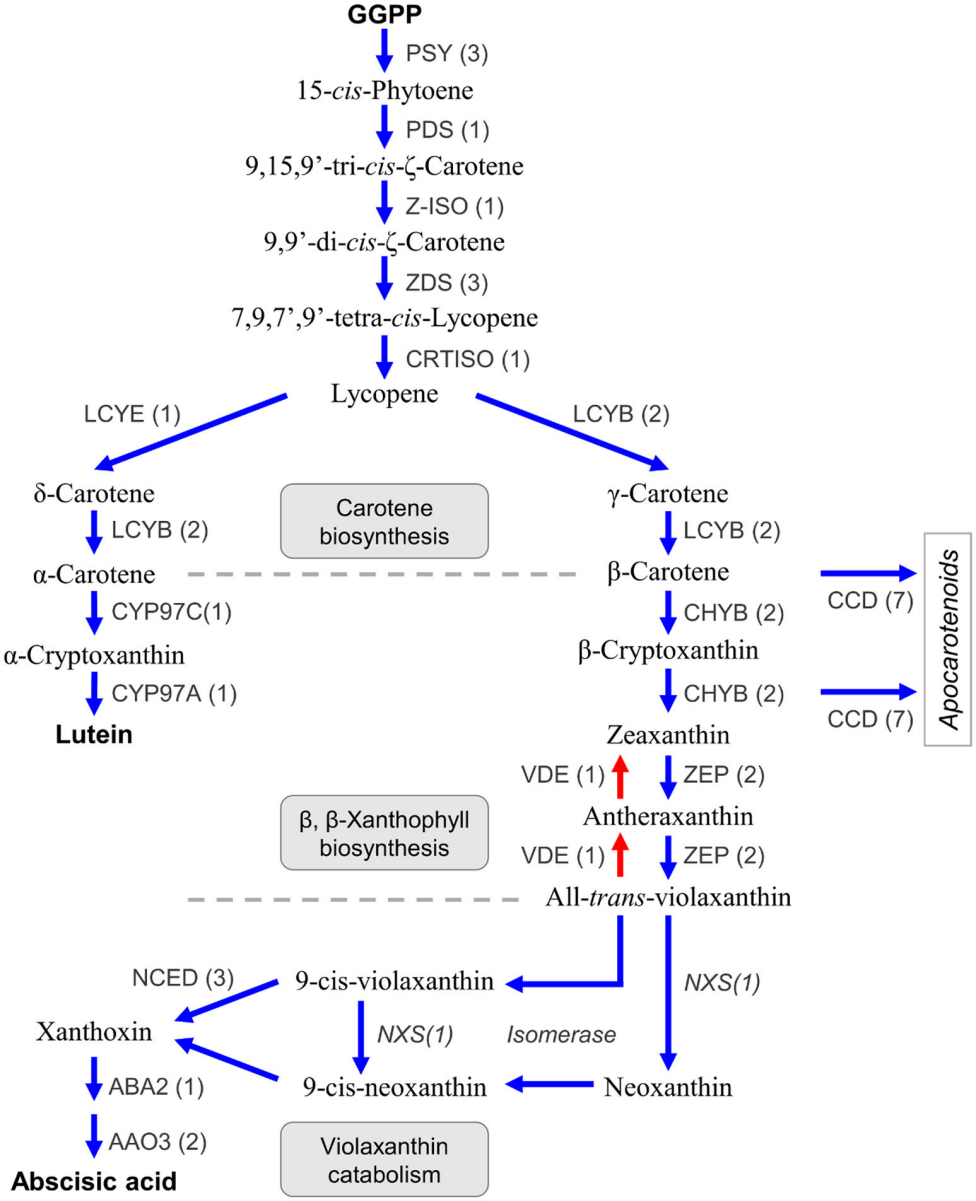
Genes involved in the biosynthesis of carotenoids and abscisic acid in Satsuma. GGPP, geranylgeranyl diphosphate; PSY, phytoene synthase; PDS, phytoene desaturase; Z-ISO, 15-cis-zeta-carotene isomerase; ZDS, zeta-carotene desaturase; CRTISO, carotenoid isomerase/prolycopene isomerase; LCYE, lycopene epsilon-cyclase; LCYB, lycopene beta-cyclase; CYP97C, carotene epsilon-monooxygenase; CYP97A, beta-carotene 3-hydroxylase; CHYB, beta-carotene 3-hydroxylase; ZEP, zeaxanthin epoxidase; VDE, violaxanthin de-epoxidase; CCD, carotenoid cleavage dioxygenase; NCED, 9-cis-epoxycarotenoid dioxygenase; NXS, Neoxanthin synthase, putative; ABA, xanthoxin dehydrogenase; AAO, abscisic-aldehyde oxidase. Numbers in parentheses represent the number of each detected gene.

Carotenoids are a class of diterpenoid compounds contained in plants as orange, yellow, or red color pigments that are important in the commercial value of citrus fruit because they attract consumers. Carotenoids in dietary intake serve as an antioxidants (Fraser and Bramley, 2004) and recent studies suggested the carotenoid compound β-cryptoxanthin was particularly abundant in the fruit of Satsuma, thus providing health benefits that decrease the risk of serum lipid levels, metabolic syndrome, and osteoporosis (Sugiura et al., 2004, 2015, 2016). Furthermore, carotenoids are the substrate from which the plant hormone abscisic acid (Kato et al., 2006; Alquézar et al., 2008) and strigolactone (Bruno and Al-Babili, 2016) are synthesized and they also function in protection from excess light irradiation in the photosynthetic apparatus by light acclimation within the xanthophyll cycle (Jahns and Holzwarth, 2012). Three types of carotenoid compounds, lineal (phytoene, lycopene), bicyclic (carotene), and epoxy carotenoid (violaxanthin) are synthesized from GGPP as the substrate in a stepwise manner by branched biosynthesis pathways, in plastids (Figure 6) (Fraser and Bramley, 2004; Yuan et al., 2015). Their compositions and contents in the rind or juice sac differ among varieties (Matsumoto et al., 2007) and change during fruit maturation by regulation at different levels (Kato et al., 2004; Alquézar et al., 2008). Gene mining of the predicted gene identified seven genes for carotene biosynthesis from GGPP (Figure 6, Table S8). The number of genes for carotene biosynthesis (phytoene synthase, PSY; phytoene desaturase, PDS; ς-carotene desaturase, ZDS; and lycopene β-cyclase, LCYB) were consistent with those reported for Satsuma (Fanciullino et al., 2007). A single copy of the genes for two isomerases [Z-ISO (Chen et al., 2010) and CRTISO Yu et al., 2011] that catalyze phytoene to lycopene were deduced. Expression of lycopene ε-cyclase (LCYE) regulates the ratio of α-carotene/β-carotene in the fruit, along with lycopene β-cyclase (LCYB). A recent study suggested there are two copies of LCYE genes in the mandarin (Fanciullino et al., 2007); however, only one copy of the LCYE gene was detected in Satsuma (Figure 6, Table S8).

Two copies of the two genes in the β,β-xanthophyll biosynthesis (β-carotene 3-hydroxylase, CHYB; and zeaxanthin epoxidase, ZDS) that regulate carotenoid composition in citrus fruits (Kato et al., 2004) were detected, which was in agreement with recent studies on CHYB (Fanciullino et al., 2007) and ZEP (Sugiyama et al., 2010). Other genes in this step (violaxanthin de-epoxidase, VDE, contributes to light acclimation (Jahns and Holzwarth, 2012); carotene ε-monooxygenase, CYP97C; and another β-carotene 3-hydroxylase, CYP97A) were detected as single copy genes (Figure 6, Table S8). Carotenoid cleavage dioxygenases (CCDs) are a heterozygous gene family that consists of CCDs and 9-cis-epoxycarotenoid dioxygenases (NCEDs). Gene mining located seven CCD genes with four types (CCD1, CCD4, CCD7, and CCD8) that catalyze the synthesis of apocarotenoid compounds from β-carotene, β-cryptoxanthin, or zeaxanthin (Yuan et al., 2015). Ma et al. (2013) reported the role of CCD4 for the production of β-citraurin in citrus fruits (Ma et al., 2013). Although whole genes for violaxanthin catabolism have not been found in citrus, three types of NCED genes (NCED3, NCED5, and NCED6) that catalyze the production of xanthoxin from 9-cis-violaxanthin or 9-cis-neoxanthin, were found. Xanthoxin is a precursor of the plant hormone abscisic acid and genes that catalyze its production (xanthoxin dehydrogenase, ABA2; and abscisic-aldehyde oxidase, AAO4), were detected.

Conversely, a gene for neoxanthin synthase (NXS) or an isomerase to produce 9-cis-violaxanthin or 9-cis-neoxanthin from violaxanthin, have not been identified in citrus. However, an LCYB gene (Ciunshiu_m21164) exhibited significant similarity (*E*-value = 0.0) with a known NXS gene (Q9M424 in UniProt). Bouvier et al. (2000) reported that the NXS gene was paralogous to the LCYB gene in tomato (Bouvier et al., 2000), and the LCYB gene of Satsuma could be a primary candidate for the NXS gene in citrus. Producing xanthoxin requires isomerization of the substrate. Although their roles in xanthoxin production are still unconfirmed, the genes for three types of isomerases [Z-ISO (Chen et al., 2010), CRTISO (Yu et al., 2011) and D27 (Jamil et al., 2012)] were identified in this study. These assignments suggested that the predicted genes in Satsuma were consistent with the known gene sets for the biosynthesis and catabolism of carotenoid compounds; additionally, they may be genes encoding uncharacterized pathways.

### Genome-wide pedigree analysis for trio genotypes

**Figure 7:**
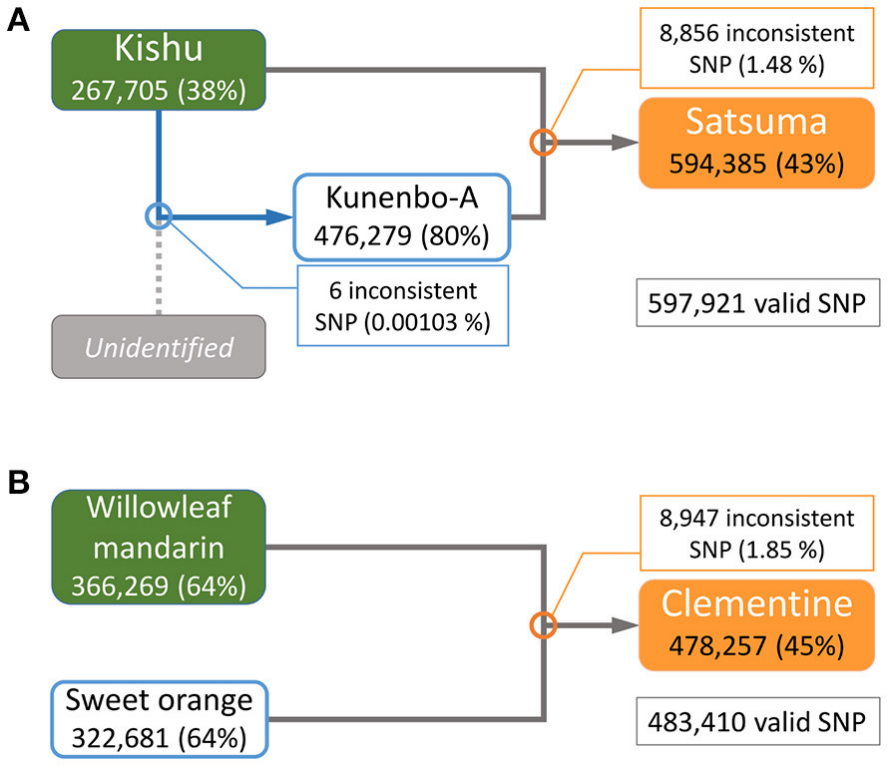
Genome-wide parentage analysis of Satsuma and Clementine trios. **(A)** The pedigree of Satsuma as an offspring of Kishu (seed parent) and kunenbo-A (pollen parent). Kunenbo-A was also considered to be an offspring of Kishu, with an unidentified variety as the seed parent (Shimizu et al., 2016b). **(B)** The pedigree of the Clementine as the offspring of Willowleaf mandarin (seed parent) and sweet orange (pollen parent). The numbers under the variety name in rounded rectangles are heterozygous SNPs for each, and the % ratio in the parentheses are the site coverage ratios of the detected variant sites to all variant sites. The numbers in the rectangles represent the number of SNPs that were inconsistent among the trio by parentage analysis at each cross pointed with circles, and the numbers in parentheses are the ratios of the inconsistent SNP sites to the valid SNPs.

The parentage of Satsuma as an offspring of Kishu (*C. kinokuni* hort. ex Tanaka) and kunenbo-A (*C. nobilis* Lour. var. kunep Tanaka) (Shimizu et al., 2016b) was confirmed by pedigree analysis of this trio with their genome-wide genotypes. Resequencing analysis of the short-reads to the assembled genome sequence detected 3,478,214 variant sites in the trio for Satsuma and 597,921 SNP sites were selected without failure of data acquisition (NA) among the three varieties. A similar resequencing analysis for the Clementine with the parent varieties (Willowleaf mandarin and sweet orange) (Wu et al., 2014; Shimizu et al., 2016b) identified 3,725,026 variant sites, and 483,410 SNP sites were selected as valid for these three varieties (Figure 7). A parentage analysis detected 8,856 SNP (1.48%) sites that were inconsistent with the Satsuma trio (Figure 7). Another parentage analysis showed that 8,947 (1.85%) sites were inconsistent with the Clementine trio and it was slightly higher than that found for the Satsuma trio. Since other genome sequencing efforts of various Satsuma strains found a considerable number of polymorphisms at various sites (Shimizu et al., in preparation), the inconsistent SNP genotypes could be caused by spontaneous mutations that occurred during the extended vegetative propagation period of these trees. Furthermore, kunenbo-A and Kishu shared allele in all SNP sites, except six, and confirmed a fact which the proposed parentage of kunenbo-A as an offspring of Kishu (Shimizu et al., 2016b) (Figure 7). These analyses confirmed the proposed parentages of Satsuma as an back-crossed offspring of Kishu and suggested that the accuracy of the assembled sequence constructed by the hybrid assembly approach was high enough to examine genome-wide parentage analysis.

### Data availability

The assembled draft genomic sequences are deposited in the DNA Data Bank of Japan (DDBJ) with accession numbers from BDQV01000001 to BDQV01020876. The assembled sequence with accompanying data are available at http://www.citrusgenome.jp.

## Discussion

Using a haploid or doubled haploid plant is the “gold standard” in the production of a high-quality genome sequence for heterozygous fruit tree varieties (Patel et al., 2015; Daccord et al., 2017). To date, three reference genome sequences of citrus have been published, for Clementine (Wu et al., 2014), pummelo and sweet orange (Xu et al., 2013; Wang et al., 2017), which were constructed with haploid or doubled haploid plants. Resequencing analysis based on the reference genome sequence is a quick and cost-effective approach to reveal genetic diversity and mining polymorphic loci among closely related varieties. However, the wide genetic diversity within citrus varieties would cause the resequencing approach to fail at highly divergent regions among the varieties.

Along with the dramatic drop in the cost of NGS analysis over the past decade, *de novo* genome assembly of various varieties has facilitated genome-wide DNA marker development and gene identification. Furthermore, citrus varieties for which genome sequencing development succeeded for the haploid or doubled haploid plants are still limited to particular varieties, such as the Clementine, sweet orange, Lee mandarin, and trifoliate orange (Germana, 2007) whereas there is no report for Satsuma, which is one of the most important mandarin varieties in the world. In this study, we applied a hybrid assembly approach which integrated short-read sequences, three mate-pair libraries of 3, 5, and 8 kb of insert sizes, and a long-read sequence in a stepwise manner for the assembly of the heterozygous diploid genome of Satsuma.

Initial assembly and scaffolding with short-read sequences and the three mate-pair libraries by the PLATANUS assembler (Kajitani et al., 2014) that focused on heterozygous genome assembly produced a 348 Mb preliminary sequence consisting of 20,973 scaffolds. The finishing step with long-read sequence obtained from PacBio did not greatly alter the number of scaffolds or total length, but it did effectively decrease the ratio of indeterminate nucleotides from 12.5 to 7.9%. Finally, it produced a *de novo* draft sequence of the Satsuma genome of 359,652,061 bp consisting of 20,876 scaffolds and N_50_ length of 386,404 bp (Table 1).

We applied several strategies to evaluate the quality of the assembled sequence. The BUSCO scores for the assembled scaffolds (Table 2) and the distance between the mapped BAC end sequences (Table 3) suggested that the consistency of the assembled sequence was comparable to the haploid genomes. Other BUSCO scores suggested that the accuracy of gene prediction was close to those of the haploid genomes (Table 2). The ratios and composition of repeat elements, LTR retrotransposon and simple sequence repeat (SSR) also confirmed the quality of the assembled sequence and suggested no obvious problems in the assembly at the repeat region. Furthermore, the pseudomolecule of Satsuma revealed genome-wide synteny to other citrus genomes (Table 4, Figure 2). The total length of the obtained pseudomolecule was not enough, but a further extension will be attained by aligning it with a high-density linkage map that is consisted of more DNA markers. These results indicated the effectiveness of PLATANUS for assembling the heterozygous genome from short-read and mate-pair libraries and confirmed the validity of the hybrid approach for improving sequence quality by filling indeterminate nucleotides with the long-read sequence. The assembled genome size was close to those of the previously reported size (Ollitrault et al., 1994), but the estimated genome sizes of Satsuma by *k-mer* analysis were smaller than the reported size. No clear relationship between estimated genome size and their heterozygosity was indicated. The reason for such significant discrepancy was not clear, but repeat element that accounts for about 40% of the genome (Table 5) may interfere with size estimation.

The number of predicted genes and mRNA by MAKER-P (Campbell et al., 2014) were slightly higher than those of Clementine (Wu et al., 2014) but close to those of pummelo (Wang et al., 2017). Functional annotations identified the genes for each clan of the P450 (CYP) family and covered all members of the known CYP gene family in citruses. The numbers of genes in each clan were close to those of the genes in Clementine or sweet orange reported recently (Mittapelli et al., 2014). Furthermore, unknown genes for the key enzymes of gibberellin biosynthesis that regulate gibberellin homeostasis in plant tissues (GA20ox, GA2ox, and GA3ox) were identified from functional annotation analysis, which also identified candidate genes for the early 13-hydroxylation pathway (GA13ox, putative). Because gibberellins contribute to strong parthenocarpy of Satsuma (Talón et al., 1990; Ben-Cheikh et al., 1997), further functional analysis of these genes is anticipated to contribute to breeding for higher parthenocarpy varieties. Likewise, genes assigned to the biosynthesis of carotenoids and abscisic acid consisted of the known set for each step in these pathways. Although genes for catalysis of xanthoxin were not identified in *Citrus*, a candidate gene for neoxanthin synthesis (NXS, neoxanthin synthase) was first proposed in this study. Future functional analysis of this gene will clarify the process of violaxanthin catabolism and abscisic acid synthesis. Another application of the assembled sequence confirmed the parentage of Satsuma with a comparable score of inconsistent SNP sites to that of the Clementine trio and confirmed Satsuma is a back-crossed offspring of Kishu (Figure 7).

In conclusion, the hybrid assembly approach is a quick and reliable method for developing a draft sequence for a heterozygous genome that is sufficient for gene discovery study and genome-wide parentage analysis.

## Author contributions

TS, YT, EK and YN designed the study. TY and TS maintained and prepared the plant materials. AT, AF, EK, YN and TS prepared sequencing libraries and conducted sequencing. YT, EK, TM, HN, YN and TS assembled and evaluated the draft sequence for repeat elements, quality evaluation, and synteny to the reference genome. YT, TS, and EK conducted gene prediction and functional annotation. EK and TS conducted the parentage analysis. TS, YT, and EK drafted the manuscript. All authors contributed to the manuscript.

### Conflict of interest statement

The authors declare that the research was conducted in the absence of any commercial or financial relationships that could be construed as a potential conflict of interest.
