# Supplementary material for: Draft Sequencing of the Heterozygous Diploid Genome of Satsuma (Citrus unshiu Marc.) Using a Hybrid Assembly Approach
Source: Front Genet. 2017 Dec 5;8:180. doi: 10.3389/fgene.2017.00180 (PMC5723288; doi:10.3389/fgene.2017.00180)
Supplement: Supplementary file 1 [file Table1.PDF]

Shimizu, T. et al (2017) Draft sequencing of the heterozygous diploid genome of Satsuma (*Citrus unshiu* Marc.) using a hybrid assembly approach

Supplemental Table S1 NGS reads used for the assembly of Satsuma genome

Illumina HiSeq 2000:

Reads libraries	Reads	Reads after trim	Passed reads (%)	Avg. size after trim (bp)	Coverag e
Paired end (300 bp)	431,239,126	298,902,618	69.3	100	X 82.8
Mate pair (3 kbp)	147,549,796	64,815,094	43.9	98.6	X 17.5
Mate pair (5 kbp)	157,408,396	90,124,484	57.3	98.8	X 24.7
Mate pair (8 kbp)	142,384,720	39,113,324	27.5	98	X 10.6

PacBio RS II:

run = 6	Reads	Length (bp)	Average size (bp)	Frequent size	Coverage
Subreads	1,174,045	2,838,383,648	2,418	1,400-1,800 bp	X 7.88
CCS reads	78,300	155,964,679	1,992	1,400-1,800 bp	X 0.43
