# Supplementary material for: Draft Sequencing of the Heterozygous Diploid Genome of Satsuma (Citrus unshiu Marc.) Using a Hybrid Assembly Approach
Source: Front Genet. 2017 Dec 5;8:180. doi: 10.3389/fgene.2017.00180 (PMC5723288; doi:10.3389/fgene.2017.00180)
Supplement: Supplementary file 2 [file Table2.PDF]

Shimizu, T. et al (2017) Draft sequencing of the heterozygous diploid genome of Satsuma (*Citrus unshiu* Marc.) using a hybrid assembly approach

Supplemental Table S2 Summary of the estimated repeat elements

Total genome length:			359,652,061 bp	
bases masked:			142,143,773 bp	39.52 %
Type of repeat		Number of elelemts [1]	Length occupied	% of sequence
SINEs:		0	0 bp	0 %
	ALUs	0	0 bp	0 %
	MIRs	0	0 bp	0 %
LINEs:		5,720	5,183,751 bp	1.44 %
	LINE1	4,473	4,768,340 bp	1.33 %
	LINE2	307	177,740 bp	0.05 %
	L3/CR1	74	83,680 bp	0.02 %
LTR elements:		90,807	77,615,313 bp	21.58 %
	ERVL	0	0 bp	0 %
	ERVL-MaLRs	0	0 bp	0 %
	ERV_classI	0	0 bp	0 %
	ERV_classII	40	27,290 bp	0.01 %
DNA elements:		49,986	18,536,002 bp	5.15 %
	hAT-Charlie	0	0 bp	0 %
	TcMar-Tigger	0	0 bp	0 %
Unclassified:		94,706	35,171,027 bp	9.78 %
Total interspersed repeats:			136,506,093 bp	37.95 %
Small RNA:		0	0 bp	0 %
Satellites:		71	61,151 bp	0.02 %
Simple repeats:		107,945	5,189,492 bp	1.44 %
Low complexity:		20,855	1,011,154 bp	0.28 %

[1] Estimated repeats that were fragmented by insertions or deletions were counted as one element.
