# Supplementary material for: Draft Sequencing of the Heterozygous Diploid Genome of Satsuma (Citrus unshiu Marc.) Using a Hybrid Assembly Approach
Source: Front Genet. 2017 Dec 5;8:180. doi: 10.3389/fgene.2017.00180 (PMC5723288; doi:10.3389/fgene.2017.00180)
Supplement: Supplementary file 3 [file Table3.PDF]

Shimizu, T. et al (2017) Draft sequencing of the heterozygous diploid genome of Satsuma (*Citrus unshiu* Marc.) using a hybrid assembly approach

Supplemental Table S3 Repeat elements detected with RepeatMasker

Repeat elements:		Number of elements	seq length (bp)	% of sequenc	
DNA elements:		55,163	19,086,450	5.31	%
	DNA	3,962	780,344	0.22	%
	DNA/CMC-EnSpm	5,109	2,385,616	0.66	%
	DNA/CMC-Transib	940	309,680	0.09	%
	DNA/Ginger	416	124,654	0.03	%
	DNA/MULE-MuDR	8,825	3,949,734	1.10	%
	DNA/Maverick	278	103,436	0.03	%
	DNA/PIF-Harbinger	6,595	2,034,942	0.57	%
	DNA/Sola-1	149	79,172	0.02	%
	DNA/TcMar-Pogo	2,930	617,239	0.17	%
	DNA/hAT-Ac	20,428	6,657,781	1.85	%
	DNA/hAT-Tip100	5,531	2,043,852	0.57	%
LINE:		7,185	5,203,552	1.45	%
	LINE/CR1	105	83,757	0.02	%
	LINE/I-Jockey	170	57,159	0.02	%
	LINE/L1	5,732	4,774,180	1.33	%
	LINE/L2	422	181,501	0.05	%
	LINE/Tad1	756	106,955	0.03	%
LTR:		107,846	78,165,620	21.73	%
	LTR	1,252	329,470	0.09	%
	LTR/Caulimovirus	12,111	9,081,208	2.52	%
	LTR/Copia	51,986	33,091,609	9.2	%
	LTR/ERVK	57	27,309	0.01	%
	LTR/Gypsy	41,811	35,351,573	9.83	%
	LTR/Ngaro	629	284,451	0.08	%
	Low_complexity	21,045	1,013,551	0.28	%
	RC/Helitron	3,559	1,879,465	0.52	%
	Satellite	78	61,228	0.02	%
	Simple_repeat	108,854	5,262,386	1.46	%
Unclassified:	Unknown	104,621	34,814,659	9.68	%

The fragmented repeats were counted separately in this table.
