# Supplementary material for: Draft Sequencing of the Heterozygous Diploid Genome of Satsuma (Citrus unshiu Marc.) Using a Hybrid Assembly Approach
Source: Front Genet. 2017 Dec 5;8:180. doi: 10.3389/fgene.2017.00180 (PMC5723288; doi:10.3389/fgene.2017.00180)
Supplement: Supplementary file 4 [file Table4.PDF]

Shimizu, T. et al (2017) Draft sequencing of the heterozygous diploid genome of Satsuma (*Citrus unshiu* Marc.) using a hybrid assembly approach

Supplemental Table S4 Predicted gene families sorted according to similarity search to the curated cDNA database of Arabidopsis

#	Super gene families	Gene families	Classified genes	Ratio (%)
1	ABC Superfamily	17	294	3.66%
2	Acyl Lipid Metabolism Family	163	562	6.99%
3	AGC Family	7	27	0.34%
4	Aldehyde dehydrogenase (ALDH) superfamily	9	20	0.25%
5	Antiporters	12	80	1.00%
6	Aquaporin Families	1	25	0.31%
7	C2H2 zinc finger proteins	1	11	0.14%
8	Carbohydrate Esterase Gene Families	5	83	1.03%
9	Chloroplast and Mitochondria gene families	16	55	0.68%
10	Cytochrome b5	2	11	0.14%
11	Cytochrome P450	57	341	4.24%
12	Cytoplasmic ribosomal protein gene family	77	221	2.75%
13	Cytoplasmic	1	1	0.01%
14	Cytoskeleton	2	10	0.12%
15	Expansins	4	31	0.39%
16	F-Box Proteins	1	3	0.04%
17	FH2 proteins	2	15	0.19%
18	FtsH: AAA ATP-dependent zinc metallopeptidase	1	11	0.14%
19	GAGA-motif binding transcription factor familiy	2	3	0.04%
20	Glycoside Hydrolase Gene Families	25	274	3.41%
21	Glycosyltransferase Gene Families	27	324	4.03%
22	GST superfamily	7	79	0.98%
23	Heat Shock Transcription Factors	1	20	0.25%
24	Homeodomain protein	1	11	0.14%
25	Inorganic Solute Cotransporters	14	89	1.11%
26	Ion Channel Families	6	85	1.06%
27	Kinesins	1	54	0.67%
28	Leucine-rich repeat extensin	3	16	0.20%
29	Lipid Metabolism Gene Families	27	71	0.88%
30	Longin Group	4	14	0.17%
31	MAPKKK	3	79	0.98%
32	Monolignol Biosynthesis	13	161	2.00%
33	Monosaccharide transporter-like gene family	7	58	0.72%
34	MYB	1	107	1.33%
35	Myosin	1	10	0.12%
36	NADPH P450 reductases	1	3	0.04%
37	NSF and SNAP Proteins	3	3	0.04%
38	NULL	220	3,817	47.48%
39	Organic Solute Cotransporters	32	293	3.64%
40	Other SNAREs	4	8	0.10%
41	Phospholipase D	1	10	0.12%
42	Phosphoribosyltransferases (PRT)	4	21	0.26%
43	Plant Cell Wall Biosynthesis Families	6	41	0.51%
44	Plant defensins superfamily	1	1	0.01%
45	Polysaccharide Lyase Gene Families	2	25	0.31%
46	PP2C-type phosphatases	11	73	0.91%

47	Primary Pumps (ATPases) Gene Families	12	94	1.17%
48	Protein synthesis factors	39	97	1.21%
49	Response Regulator	3	23	0.29%
50	RRE (rapid response to elicitors)	1	5	0.06%
51	SEC1 Proteins	4	8	0.10%
52	SNAP25-like group	1	2	0.02%
53	Sulfurtransferasese / Rhodanese Family	6	17	0.21%
54	Superfamily of zinc-coordinating DNA-binding proteins	1	14	0.17%
55	Syntaxin	10	25	0.31%
56	Transcription Factor	1	128	1.59%
57	Transporter Superfamily (uniporter, symporter, antiporte	1	5	0.06%
58	Trehalose Biosynthesis Gene Families	2	8	0.10%
59	WRKY Transcription Factor Superfamily	7	51	0.63%
60	Zinc finger-homeobox gene family	2	12	0.15%
	Total	896	8,040	
