# Supplementary material for: Draft Sequencing of the Heterozygous Diploid Genome of Satsuma (Citrus unshiu Marc.) Using a Hybrid Assembly Approach
Source: Front Genet. 2017 Dec 5;8:180. doi: 10.3389/fgene.2017.00180 (PMC5723288; doi:10.3389/fgene.2017.00180)
Supplement: Supplementary file 5 [file Table5.PDF]

Shimizu, T. et al (2017) Draft sequencing of the heterozygous diploid genome of Satsuma (*Citrus unshiu* Marc.) using a hybrid assembly approach

Supplemental Table S5 Summary of the primary transcripts assigned to protein families

Protein families: metabolism	Top classes	Second classes	Assigned Transcripts	Assgined to map
Enzymes	62	175	4,507	1,628
Protein kinases	8	49	407	197
Protein phosphatases and associated proteins	3	7	176	30
Peptidases	5	47	266	43
Glycosyltransferases	7	20	234	36
Lipopolysaccharide biosynthesis proteins	3	0	8	12
Lipid biosynthesis proteins	4	9	83	51
Prenyltransferases	3	11	56	35
Amino acid related enzymes	2	11	86	79
Cytochrome P450	2	34	80	19
Photosynthesis proteins	2	3	45	6
Total	101	366	5,948	2,136

Protein families: genetic information processing	Top classes	Second classes	Assigned Transcripts	Assgined to map
Transcription factors	2	7	345	391
Transcription Machinery	2	5	285	70
Messenger RNA biogenesis	2	5	458	173
Spliceosome	5	14	541	300
Ribosome	1	4	536	514
Ribosome biogenesis	1	1	6	0
Transfer RNA biogenesis	2	10	246	97
Translation factors	1	2	74	11
Chaperones and folding catalysts	1	1	25	3
Membrane trafficking	8	32	629	83
Ubiquitin system	1	1	138	79
Proteasome	1	1	27	23
DNA replication proteins	2	2	32	26
Chromosome and associated proteins	1	1	6	0
DNA repair and recombination proteins	2	3	47	35
Mitochondrial biogenesis	3	12	328	48
Total	35	101	3,723	1,853

Protein families: signaling and cellular processes	Top classes	Second classes	Assigned Transcripts	Assgined to map
Secretion system proteins	3	5	36	22
Cytoskeleton proteins	1	1	4	0
Exosome	1	12	788	207
Prokaryotic defense system	2	2	10	8
G Protein-Coupled Receptors	1	1	12	0
Ion Channels	4	8	57	0
GTP-binding proteins	2	6	72	3
CD molecules	1	0	27	0
Glycosaminoglycan binding proteins	2	4	10	4
Glycosylphosphatidylinositol (GPI)-anchored proteins	3	0	11	0
Total	20	39	1,027	244
