# Supplementary material for: Draft Sequencing of the Heterozygous Diploid Genome of Satsuma (Citrus unshiu Marc.) Using a Hybrid Assembly Approach
Source: Front Genet. 2017 Dec 5;8:180. doi: 10.3389/fgene.2017.00180 (PMC5723288; doi:10.3389/fgene.2017.00180)
Supplement: Supplementary file 6 [file Table6.PDF]

Shimizu, T. et al (2017) Draft sequencing of the heterozygous diploid genome of Satsuma (*Citrus unshiu* Marc.) using a hybrid assembly approach

Supplemental Table S6 Summary of the predicted protein coding genes assigned to the metabolic pathways based on the KEGG module database

**Pathway module (26 categories)**	Modules	Genes	Assigned
Energy metabolism	13	144	144
Nitrogen metabolism	1	2	2
Methane metabolism	4	27	27
Sulfur metabolism	2	8	8
Carbohydrate and lipid metabolism	15	250	250
Other carbohydrate metabolism	20	288	288
Lipid metabolism	12	91	91
Lipopolysaccharide metabolism	3	9	7
Glycan metabolism	7	47	47
Glycosaminoglycan metabolism	2	13	13
Terpenoid backbone biosynthesis	6	51	51
Sterol biosynthesis	4	15	15
Other terpenoid biosynthesis	3	31	31
Nucleotide and amino acid metabolism	4	65	65
Pyrimidine metabolism	4	33	33
Serine and threonine metabolism	3	15	15
Cysteine and methionine metabolism	6	69	69
Branched-chain amino acid metabolism	5	50	50
Lysine metabolism	6	36	36
Arginine and proline metabolism	3	16	16
Histidine metabolism	1	9	9
Aromatic amino acid metabolism	8	79	79
Other amino acid metabolism	2	5	5
Cofactor and vitamin biosynthesis	21	153	153
Polyamine biosynthesis	3	19	19
Secondary metabolism	4	124	124
Sub total	162	1,649	1,647

**Structural complex module (13 categories)**	Modules	Genes	Assigned to map
Energy metabolism	15	122	122
Genetic information processing	4	14	14
Replication system	5	32	28
Repair system	8	61	61
RNA polymerase	4	54	54
Spliceosome	9	188	188
RNA processing	13	126	126
Ribosome	3	429	429
Proteasome	3	59	59
Ubiquitin system	13	172	172
Protein processing	8	69	69
Saccharide, polyol, and lipid transport system	1	1	0
Bacterial secretion system	3	13	12
Sub total	89	1,340	1,334

**Functional set module (three categories)**	Modules	Genes	Assigned to map
Metabolism	4	130	130
Environmental information processing	2	8	0
Cellular processes	12	247	247
Sub total	18	385	377

**Signature module (one category)**	Modules	Genes	Assigned to map
Gene set	2	25	0

Total	271	3,399	3,358
