# Supplementary material for: Draft Sequencing of the Heterozygous Diploid Genome of Satsuma (Citrus unshiu Marc.) Using a Hybrid Assembly Approach
Source: Front Genet. 2017 Dec 5;8:180. doi: 10.3389/fgene.2017.00180 (PMC5723288; doi:10.3389/fgene.2017.00180)
Supplement: Supplementary file 7 [file Table7.PDF]

Shimizu, T. et al (2017) Draft sequencing of the heterozygous diploid genome of Satsuma (*Citrus unshiu* Marc.) using a hybrid assembly approach

Supplemental Table S7 Deduced cytochrome P450 genes of Satsuma sorted to their clans and families

CYP clan	CYP family	Gene ID	CYP name	Evidence	E-value
CYP 71 clan					
	CYP71A	Ciunshiu_m20485	CYP71A1	U	1E-131
	CYP71A	Ciunshiu_m03124	CYP71A1	U	1E-103
	CYP71A	Ciunshiu_m03127	CYP71A1	U	1E-130
	CYP71A	Ciunshiu_m07969	CYP71A1	U,K	1E-117
	CYP71A	Ciunshiu_m07987	CYP71A1	U	1E-130
	CYP71A	Ciunshiu_m23916	CYP71A1	U	1E-103
	CYP71A	Ciunshiu_m25798	CYP71A1	U	1E-97
	CYP71A	Ciunshiu_m25788	CYP71A1	U	1E-38
	CYP71A	Ciunshiu_m00795	CYP71A1	U	5E-88
	CYP71A	Ciunshiu_m06939	CYP71A1	U	1E-144
	CYP71A	Ciunshiu_m07013	CYP71A1	U	1E-172
	CYP71A	Ciunshiu_m19729	CYP71A1	U	1E-164
	CYP71A	Ciunshiu_m06940	CYP71A20	A,U	4E-89
	CYP71A	Ciunshiu_m08668	CYP71A20	A,U	1E-121
	CYP71A	Ciunshiu_m06028	CYP71A21	U	1E-151
	CYP71A	Ciunshiu_m16350	CYP71A26	U	1E-135
	CYP71A	Ciunshiu_m07004	CYP71A9	K,U	1E-156
	CYP71B	Ciunshiu_m00236	CYP71B10	A	7E-48
	CYP71B	Ciunshiu_m03353	CYP71B14	A	6E-79
	CYP71B	Ciunshiu_m00801	CYP71B2	A,U	1E-92
	CYP71B	Ciunshiu_m05948	CYP71B23	A,U	7E-54
	CYP71B	Ciunshiu_m10454	CYP71B23	A,U	1E-110
	CYP71B	Ciunshiu_m10472	CYP71B23	A,U	1E-109
	CYP71B	Ciunshiu_m11908	CYP71B30P	A	7E-34
	CYP71B	Ciunshiu_m09976	CYP71B34	A,U	1E-101
	CYP71B	Ciunshiu_m14096	CYP71B34	A,U	2E-92
	CYP71B	Ciunshiu_m17923	CYP71B34	A	4E-59
	CYP71B	Ciunshiu_m01658	CYP71B35	A,U	1E-147
	CYP71B	Ciunshiu_m09243	CYP71B35	A,U	2E-71
	CYP71B	Ciunshiu_m20557	CYP71B35	A,U	1E-140
	CYP71B	Ciunshiu_m00659	CYP71B7	A,U	1E-122
	CYP71B	Ciunshiu_m18426	CYP71B9	U	1E-130
	CYP71D	Ciunshiu_m20793	CYP71D11	U	0
	CYP71D	Ciunshiu_m10473	CYP71D11	U	0
	CYP71D	Ciunshiu_m10455	CYP71D12	U	1E-95
	CYP71D	Ciunshiu_m09974	CYP71D55	U	1E-167
	CYP71D	Ciunshiu_m02403	CYP71D55	U	1E-115
	CYP71D	Ciunshiu_m08644	CYP71D55	U	1E-162
	CYP71D	Ciunshiu_m21559	CYP71D8	U	1E-149
	CYP71D	Ciunshiu_m04767	CYP71D9	U	3E-73
	CYP73A	Ciunshiu_m12588	CYP73A5	A,U,K	1E-142
	CYP73A	Ciunshiu_m14415	CYP73A5	A,U,K	0
	CYP75A	Ciunshiu_m24220	CYP75A3	U,K	0

CYP75B	Ciunshiu_m07988	CYP75B1	A	7E-62
CYP75B	Ciunshiu_m17048	CYP75B1	A,U	1E-110
CYP75B	Ciunshiu_m25026	CYP75B1	A,U,K	0
CYP75B	Ciunshiu_m11906	CYP75B2	U	1E-123
CYP75B	Ciunshiu_m12827	CYP75B2	U	1E-132
CYP76A	Ciunshiu_m19330	CYP76A2	U,K	0
CYP76B	Ciunshiu_m22094	CYP76B10	U	0
CYP76B	Ciunshiu_m17316	CYP76B6	U	0
CYP76B	Ciunshiu_m19852	CYP76B6	U	3E-97
CYP76B	Ciunshiu_m02483	CYP76B6	U	3E-92
CYP76B	Ciunshiu_m03004	CYP76B6	U	1E-166
CYP76B	Ciunshiu_m03005	CYP76B6	U	0
CYP76B	Ciunshiu_m09066	CYP76B6	U	1E-137
CYP76B	Ciunshiu_m09839	CYP76B6	U	1E-126
CYP76B	Ciunshiu_m12157	CYP76B6	U	1E-109
CYP76B	Ciunshiu_m13216	CYP76B6	U	1E-134
CYP76B	Ciunshiu_m17313	CYP76B6	U	0
CYP76B	Ciunshiu_m22501	CYP76B6	U	1E-105
CYP76B	Ciunshiu_m26770	CYP76B6	U	7E-98
CYP76C	Ciunshiu_m00503	CYP76C2	A,U	2E-71
CYP76C	Ciunshiu_m22098	CYP76C2	A	1E-132
CYP76C	Ciunshiu_m02441	CYP76C4	A	2E-99
CYP76C	Ciunshiu_m19855	CYP76C4	A	2E-24
CYP77A	Ciunshiu_m04436	CYP77A3	U	0
CYP77B	Ciunshiu_m05417	CYP77B1	A,U	0
CYP78A	Ciunshiu_m18110	CYP78A10	A,U	0
CYP78A	Ciunshiu_m20548	CYP78A10	A,U	1E-148
CYP78A	Ciunshiu_m20616	CYP78A5	A,U	0
CYP78A	Ciunshiu_m25667	CYP78A6	A,U	0
CYP78A	Ciunshiu_m18374	CYP78A7	A,U,K	1E-173
CYP78A	Ciunshiu_m18679	CYP78A7	A,U,K	0
CYP78A	Ciunshiu_m07905	CYP78A9	A,U	0
CYP79A	Ciunshiu_m02975	CYP79A2	A,U,K	0
CYP79A	Ciunshiu_m02986	CYP79A2	A,U,K	0
CYP79A	Ciunshiu_m02991	CYP79A2	A,U,K	1E-168
CYP79A	Ciunshiu_m02995	CYP79A2	A,U	1E-112
CYP79A	Ciunshiu_m11589	CYP79A2	A,U,K	1E-173
CYP79A	Ciunshiu_m14637	CYP79A2	A,U	1E-159
CYP79A	Ciunshiu_m23676	CYP79A2	A,U,K	1E-156
CYP79A	Ciunshiu_m24342	CYP79A2	A,U	2E-98
CYP79A	Ciunshiu_m24931	CYP79A2	A,U,K	0
CYP79A	Ciunshiu_m24975	CYP79A2	A,U,K	0
CYP81D	Ciunshiu_m18294	CYP81D11	U	1E-109
CYP81D	Ciunshiu_m07441	CYP81D11	U	2E-54
CYP81E	Ciunshiu_m27030	CYP81E1	U	1E-155
CYP81E	Ciunshiu_m09103	CYP81E8	U	1E-127
CYP81E	Ciunshiu_m13916	CYP81E8	U	0
CYP81E	Ciunshiu_m13921	CYP81E8	U	0
CYP81E	Ciunshiu_m13917	CYP81E8	U	1E-176
CYP81E	Ciunshiu_m02459	CYP81E9	U	6E-67
CYP82A	Ciunshiu_m26182	CYP82A3	U	1E-60

CYP82A	Ciunshiu_m16923	CYP82A3	U	1E-172
CYP82A	Ciunshiu_m22847	CYP82A3	U	1E-163
CYP82A	Ciunshiu_m27021	CYP82A3	U	1E-165
CYP82C	Ciunshiu_m11081	CYP82C4	U	5E-76
CYP82C	Ciunshiu_m16922	CYP82C4	U	1E-144
CYP82C	Ciunshiu_m18183	CYP82C4	U	0
CYP82C	Ciunshiu_m02992	CYP82C4	A,U	1E-138
CYP82C	Ciunshiu_m03934	CYP82C4	A	1E-125
CYP82C	Ciunshiu_m03936	CYP82C4	A	1E-114
CYP82C	Ciunshiu_m09297	CYP82C4	A	1E-109
CYP82C	Ciunshiu_m11365	CYP82C4	A,U	1E-163
CYP82C	Ciunshiu_m21301	CYP82C4	A,U	1E-142
CYP82C	Ciunshiu_m21302	CYP82C4	A,U	1E-162
CYP82C	Ciunshiu_m22423	CYP82C4	A	5E-66
CYP82C	Ciunshiu_m22844	CYP82C4	A,U	1E-156
CYP82C	Ciunshiu_m22845	CYP82C4	A,U	1E-156
CYP82C	Ciunshiu_m25915	CYP82C4	A	2E-80
CYP82C	Ciunshiu_m27022	CYP82C4	A,U	1E-155
CYP82C	Ciunshiu_m28797	CYP82C4	A	1E-130
CYP82G	Ciunshiu_m25197	CYP82G1	A,U,K	1E-156
CYP83B	Ciunshiu_m20755	CYP83B1	U	1E-113
CYP83B	Ciunshiu_m23045	CYP83B1	U	1E-170
CYP83B	Ciunshiu_m02416	CYP83B1	U	1E-165
CYP83B	Ciunshiu_m00534	CYP83B1	U	1E-119
CYP83B	Ciunshiu_m11960	CYP83B1	U	1E-114
CYP83B	Ciunshiu_m00535	CYP83B1	A,U	3E-99
CYP83B	Ciunshiu_m00541	CYP83B1	A,U	1E-98
CYP83B	Ciunshiu_m00661	CYP83B1	A	1E-58
CYP83B	Ciunshiu_m01189	CYP83B1	A,U	1E-109
CYP83B	Ciunshiu_m07451	CYP83B1	A	5E-37
CYP83B	Ciunshiu_m09680	CYP83B1	A	2E-36
CYP83B	Ciunshiu_m11961	CYP83B1	A,U	1E-116
CYP83B	Ciunshiu_m14095	CYP83B1	A,U	5E-98
CYP83B	Ciunshiu_m18430	CYP83B1	A,U	2E-99
CYP83B	Ciunshiu_m24706	CYP83B1	A,U	1E-159
CYP84A	Ciunshiu_m05091	CYP84A1	A,U,K	0
CYP84A	Ciunshiu_m21126	CYP84A1	A,U,K	0
CYP89A	Ciunshiu_m06728	CYP89A2	A,U	1E-104
CYP89A	Ciunshiu_m10269	CYP89A2	U	0
CYP89A	Ciunshiu_m16968	CYP89A2	U	1E-133
CYP89A	Ciunshiu_m27793	CYP89A2	U	0
CYP89A	Ciunshiu_m10251	CYP89A2	U	0
CYP89A	Ciunshiu_m10259	CYP89A2	U	1E-179
CYP93A	Ciunshiu_m10857	CYP93A1	U	0
CYP93A	Ciunshiu_m10851	CYP93A1	U	0
CYP93A	Ciunshiu_m10858	CYP93A1	U	0
CYP93A	Ciunshiu_m10859	CYP93A1	U	0
CYP93B	Ciunshiu_m01454	CYP93B1	U	1E-136
CYP93B	Ciunshiu_m05834	CYP93B1	U	1E-166
CYP93D	Ciunshiu_m26698	CYP93D1	A	8E-82
CYP98A	Ciunshiu_m01237	CYP98A2	U,K	0
CYP98A	Ciunshiu_m15646	CYP98A2	U,K	0

	CYP98A	Ciunshiu_m01238	CYP98A3	A,K	1E-101
	CYP98A	Ciunshiu_m22545	CYP98A3	A,U,K	0
	CYP701A	Ciunshiu_m17925	CYP701A3	A,U	1E-114
	CYP701A	Ciunshiu_m19628	CYP701A3	A,U,K	0
	CYP701A	Ciunshiu_m22181	CYP701A3	A,U	0
	CYP703A	Ciunshiu_m25622	CYP703A2	A,U,K	0
	CYP706A	Ciunshiu_m01054	CYP706A3	A	1E-135
	CYP706A	Ciunshiu_m01061	CYP706A3	A	1E-116
	CYP706A	Ciunshiu_m01063	CYP706A3	A	1E-121
	CYP706A	Ciunshiu_m01101	CYP706A4	A	1E-128
	CYP706A	Ciunshiu_m01104	CYP706A4	A	1E-132
	CYP706A	Ciunshiu_m01107	CYP706A4	A	2E-91
	CYP706A	Ciunshiu_m02578	CYP706A4	A	1E-130
	CYP706A	Ciunshiu_m08467	CYP706A4	A	2E-99
	CYP706A	Ciunshiu_m01069	CYP706A6	A	1E-119
	CYP706A	Ciunshiu_m12959	CYP706A6	A	1E-100
	CYP706A	Ciunshiu_m18621	CYP706A7	A	1E-108
	CYP712A	Ciunshiu_m13318	CYP712A1	A	1E-155
	CYP712A	Ciunshiu_m13322	CYP712A1	A	0
	CYP712A	Ciunshiu_m13321	CYP712A2	A	1E-166
	CYP736A	Ciunshiu_m04412	CYP736A12	U	6E-85
	CYP736A	Ciunshiu_m03923	CYP736A12	U,K	0
	CYP736A	Ciunshiu_m04411	CYP736A12	U,K	0
	CYP736A	Ciunshiu_m09934	CYP736A12	U	2E-85
	CYP736A	Ciunshiu_m04410	CYP736A12	U,K	1E-180
CYP51 clan					
	CYP51A	Ciunshiu_m16948	CYP51A2	A	0
CYP72 clan					
	CYP72A	Ciunshiu_m09870	CYP72A10	A	1E-141
	CYP72A	Ciunshiu_m00749	CYP72A15	A	0
	CYP72A	Ciunshiu_m09871	CYP72A15	A	1E-99
	CYP72A	Ciunshiu_m00144	CYP72A15	U	1E-99
	CYP72A	Ciunshiu_m12707	CYP72A219	U	1E-168
	CYP72A	Ciunshiu_m00601	CYP72A219	U	0
	CYP72A	Ciunshiu_m00854	CYP72A219	U	1E-156
	CYP72A	Ciunshiu_m02732	CYP72A219	U	0
	CYP72A	Ciunshiu_m02740	CYP72A219	U	0
	CYP72A	Ciunshiu_m28119	CYP72A219	U	0
	CYP714A	Ciunshiu_m02939	CYP714A1	A,U,K	0
	CYP714A	Ciunshiu_m18898	CYP714A1	A,U,K	1E-148
	CYP714C	Ciunshiu_m17079	CYP714C2	U	1E-168
	CYP715A	Ciunshiu_m00905	CYP715A1	A	0
	CYP721A	Ciunshiu_m01455	CYP721A1	A	1E-122
	CYP721A	Ciunshiu_m11946	CYP721A1	A	1E-153
	CYP721A	Ciunshiu_m24820	CYP721A1	A	0
	CYP734A	Ciunshiu_m09530	CYP734A1	U,K	0

	CYP735A	Ciunshiu_m22745	CYP735A	A,K	0
	CYP749A	Ciunshiu_m20663	CYP749A22	A,K	1E-112
CYP74 clan					
	CYP74A	Ciunshiu_m18160	CYP74A	A,U	0
	CYP74A	Ciunshiu_m11390	CYP74A2	U,K	0
	CYP74B	Ciunshiu_m04086	CYP74B2	A,K	0
CYP85 clan					
	CYP85A	Ciunshiu_m20645	CYP85A1	A,U,K	0
	CYP85A	Ciunshiu_m06684	CYP85A1	K,U	0
	CYP87A	Ciunshiu_m12260	CYP87A3	U	1E-154
	CYP87A	Ciunshiu_m23704	CYP87A3	U	0
	CYP88A	Ciunshiu_m17107	CYP88A3	A,K	0
	CYP88A	Ciunshiu_m00290	CYP88A3	A,K	1E-149
	CYP88A	Ciunshiu_m18030	CYP88A3	A,K	1E-146
	CYP88A	Ciunshiu_m18032	CYP88A3	A,K	1E-148
	CYP90A	Ciunshiu_m16792	CYP90A1	A,U,K	0
	CYP90B	Ciunshiu_m22341	CYP90B1	A,U,K	0
	CYP90C	Ciunshiu_m22291	CYP90C1	A,K	0
	CYP90D	Ciunshiu_m08062	CYP90D1	A,U,K	1E-82
	CYP707A	Ciunshiu_m06670	CYP707A1	A,U,K	0
	CYP707A	Ciunshiu_m26263	CYP707A2	A,U,K	0
	CYP707A	Ciunshiu_m18461	CYP707A3	A,U	1E-87
	CYP707A	Ciunshiu_m00031	CYP707A4	A,U,K	0
	CYP707A	Ciunshiu_m16984	CYP707A4	A,U,K	0
	CYP707A	Ciunshiu_m18243	CYP707A4	A,K	0
	CYP716A	Ciunshiu_m00556	CYP716A1	A,K	0
	CYP716A	Ciunshiu_m04986	CYP716A1	A,K	1E-119
	CYP716A	Ciunshiu_m05398	CYP716A1	A	1E-58
	CYP716A	Ciunshiu_m07778	CYP716A1	A,K	1E-90
	CYP716A	Ciunshiu_m12601	CYP716A1	A,K	1E-117
	CYP716A	Ciunshiu_m23306	CYP716A1	A,K	1E-124
	CYP716B	Ciunshiu_m05394	CYP716B1	U	1E-111
	CYP716B	Ciunshiu_m09343	CYP716B1	U	2E-76
	CYP718	Ciunshiu_m09752	CYP718	A	0
	CYP720B	Ciunshiu_m00088	CYP720B1	U	1E-129
	CYP722A	Ciunshiu_m20221	CYP722A1	A	1E-172
	CYP722A	Ciunshiu_m22297	CYP722A1	A	7E-97
	CYP724B	Ciunshiu_m08645	CYP724B1	U,K	0
	CYP724B	Ciunshiu_m20698	CYP724B1	U,K	1E-171
CYP86 clan					
	CYP86A	Ciunshiu_m26151	CYP86A1	A,U,K	0
	CYP86A	Ciunshiu_m15709	CYP86A22	U,K	0

	CYP86A	Ciunshiu_m10758	CYP86A8	A,K	0
	CYP86B	Ciunshiu_m08322	CYP86B1	K,U	0
	CYP86C	Ciunshiu_m01647	CYP86C1	A	0
	CYP94A	Ciunshiu_m10921	CYP94A1	U,K	1E-155
	CYP94A	Ciunshiu_m14390	CYP94A1	U	1E-134
	CYP94B	Ciunshiu_m04738	CYP94B1	A,U,K	0
	CYP94B	Ciunshiu_m24896	CYP94B3	A,U,K	0
	CYP94C	Ciunshiu_m04459	CYP94C1	A,U	1E-143
	CYP94C	Ciunshiu_m19296	CYP94C1	A,U	1E-118
	CYP94C	Ciunshiu_m19812	CYP94C1	A,U	1E-103
	CYP94C	Ciunshiu_m20160	CYP94C1	A,U	0
	CYP94C	Ciunshiu_m20167	CYP94C1	A,U	1E-103
	CYP94D	Ciunshiu_m19801	CYP94D2	A	1E-117
	CYP94D	Ciunshiu_m22563	CYP94D2	A	0
	CYP96A	Ciunshiu_m13488	CYP96A1	A	1E-131
	CYP96A	Ciunshiu_m20735	CYP96A1	A	7E-77
	CYP96A	Ciunshiu_m12371	CYP96A10	A	1E-139
	CYP96A	Ciunshiu_m01640	CYP96A9	A	1E-105
	CYP96A	Ciunshiu_m12367	CYP96A9	A	1E-129
	CYP96A	Ciunshiu_m13489	CYP96A9	A	1E-138
	CYP704A	Ciunshiu_m14238	CYP704A2	A	0
	CYP704B	Ciunshiu_m23963	CYP704B1	K,U	0
	CYP704C	Ciunshiu_m27154	CYP704C1	U	0
CYP97 clan					
	CYP97A	Ciunshiu_m13985	CYP97A3	K,U	0
	CYP97B	Ciunshiu_m18975	CYP97B3	A	0
	CYP97C	Ciunshiu_m06563	CYP97C1	A	1E-107
	CYP97C	Ciunshiu_m08834	CYP97C1	A,U,K	0
	CYP97C	Ciunshiu_m08835	CYP97C1	A	1E-109
	CYP97C	Ciunshiu_m11761	CYP97C1	A	1E-133
	CYP97C	Ciunshiu_m28184	CYP97C1	A	1E-117
CYP710 clan					
	CYP710A	Ciunshiu_m21395	CYP710A1	A,U,K	0
CYP711 clan					
	CYP711A	Ciunshiu_m05093	CYP711A1	A,U,K	0
	CYP711A	Ciunshiu_m06279	CYP711A1	A,U,K	0

Evidence: each code represents the bases of estimation. K: by KEGG auto anotaion (KAAS), A; by TBLASTX search to Arabidopsis cDNA (Araport11), U; by BLASTX search to Uniprot.

E -value: TBLASTX or BLASTX score.
