# Supplementary material for: Draft Sequencing of the Heterozygous Diploid Genome of Satsuma (Citrus unshiu Marc.) Using a Hybrid Assembly Approach
Source: Front Genet. 2017 Dec 5;8:180. doi: 10.3389/fgene.2017.00180 (PMC5723288; doi:10.3389/fgene.2017.00180)
Supplement: Supplementary file 8 [file Table8.pdf]

Shimizu, T. et al (2017) Draft sequencing of the heterozygous diploid genome of Satsuma (*Citrus unshiu* Marc.) using a hybrid assembly approach

Supplemental Table S8 Genes involved in the biosynthesis of isoprenoids, gibberellic acids, carotenoids and abscisic acid

A: Genes for isoprenoid biosynthesis pathways

1. Mevalonic acid (MVA) pathway

Gene name	C. unshiu gene	No. genes	KO	Gene symbol	Evidences	*E* -value
Acetyl-CoA C-acetyltransferase (AACT)		2				
	Ciunshiu_m21572		K00626	AACT1	K,A	0
	Ciunshiu_m08604		K00626	AACT2	K,A	0
Hydroxymethylglutaryl-CoA synthase (HMGS)		2				
	Ciunshiu_m00987		K01641	HMGS	K,A	0
	Ciunshiu_m28761		K01641	HMGS	K,A	0
Hydroxymethylglutaryl-CoA reductase (HMGR)		1				
	Ciunshiu_m23818		K00021	HMGR1	K,A	0
Mevalonate kinase (MVK)		1				
	Ciunshiu_m26617		K00869	MVK	K,A	1E-119
Phosphomevalonate kinase (PMK)		1				
	Ciunshiu_m15083		K00938	PMK	A	1E-170
Diphosphomevalonate decarboxylase (MVD)		1				
	Ciunshiu_m12081		K01597	MVD2	K,A	0

2. Methylerythritol 4-phosphate (MEP) pathway

Gene name	C. unshiu gene	No. genes	KO	Gene symbol	Evidence	*E* -value
1-Deoxy-D-xylulose-5-phosphate synthase, chloroplastic (DXS)		3				
	Ciunshiu_m08256		K01662	DXS	K,A	0
	Ciunshiu_m21937		K01662	DXS	K,A	0
	Ciunshiu_m15304		K01662	DXS3	K,A	0
1-Deoxy-D-xylulose-5-phosphate reductoisomerase, chloroplastic (DXF		2				
	Ciunshiu_m07097		K00099	DXR	K,A	0
	Ciunshiu_m27574		K00099	DXR	K,A	0
2-C-Methyl-D-erythritol 4-phosphate cytidylyltransferase, chloroplastic (		1				
	Ciunshiu_m12436		K00991	MCT	K,A	1E-113
4-Diphosphocytidyl-2-C-methyl-D-erythritol kinase, chloroplastic (CMK)		1				
	Ciunshiu_m24165		K00919	CMK	K,A	1E-173
2-C-Methyl-D-erythritol 2,4-cyclodiphosphate synthase, chloroplastic (M		1				
	Ciunshiu_m15742		K01770	MCS	K,A	6E-95
(E)-4-hydroxy-3-methylbut-2-enyl-diphosphate synthase, chloroplastic (		1				
	Ciunshiu_m26467		K03526	HDS	K,A	0
4-hydroxy-3-methylbut-2-en-1-yl diphosphate reductase, chloroplastic (		1				
	Ciunshiu_m11400		K03527	HDR	K,A	0

3. IPP to terpenoid pathway

Gene name	C. unshiu gene	No. genes	KO	Gene symbol	Evidence	*E* -value
Isopentenyl-diphosphate delta-isomerase (IDI)		2				
	Ciunshiu_m10457		K01823	IDI1	A	5E-72
	Ciunshiu_m17745		K01823	IDI2	K,A	1E-144
Farnesyl diphosphate synthase (FDPS)		1				
	Ciunshiu_m10537		K00787	FDPS	K,A	0
Geranyl diphosphate synthase (GPS)		2				

Gene name	C. unshiu gene	No. genes	KO	Gene symbol	Evidence	*E*-value
	Ciunshiu_m05381		K14066	GPS1	K,A	1E-158
	Ciunshiu_m05382		K14066	GPS1	K,A	1E-145
Geranylgeranyl diphosphate synthase (GGPS)		6				
	Ciunshiu_m22624		K13789	GGPS2	A	5E-46
	Ciunshiu_m02816		K13789	GGPS6	A	5E-51
	Ciunshiu_m02914		K13789	GGPS10	K,A	3E-69
	Ciunshiu_m22634		K13789	GGPS11	K,A	1E-103
	Ciunshiu_m02815		K13789	GGPS11	K,A	7E-83
	Ciunshiu_m22637		K13789	GGPS11	K,A	1E-119

B: Genes for gibberellin biosynthesis

Gene name	C. unshiu gene	No. genes	KO	Gene symbol	Evidence	*E*-value
Ent-copalyl diphosphate synthase (CPS)		2				
	Ciunshiu_m13824		K04120	CPS1, GA1	K,A	0
	Ciunshiu_m23402		K04120	CPS1, GA1	K,A	0
Ent-kaurene synthase (KS)		1				
	Ciunshiu_m11633		K04121	KS1	K,A	0
Ent-kaurene oxidase (KO)		2				
	Ciunshiu_m19628		K04122	GA3, CYP701A3	K,A	0
	Ciunshiu_m22181		K04122	GA3, CYP701A3	A,U	0
Ent-kaurenoic acid oxidase (KAO)		4				
	Ciunshiu_m00290		K04123	KAO, CYP88A3	K,A	1E-149
	Ciunshiu_m17107		K04123	KAO, CYP88A3	K,A	0
	Ciunshiu_m18030		K04123	KAO, CYP88A3	K,A	1E-146
	Ciunshiu_m18032		K04123	KAO, CYP88A3	K,A	1E-148
Gibberellin 20-oxydase (GA20ox)		4				
	Ciunshiu_m01003		K05282	GA20OX1	K,A	1E-167
	Ciunshiu_m14253		K05282	GA20OX2	K,A	1E-132
	Ciunshiu_m06928		K05282	GA20OX	K,A	1E-103
	Ciunshiu_m24003		K05282	GA20OX	K,A	1E-116
Gibberellin 2-oxidase (GA2ox)		8				
	Ciunshiu_m12200		K04125	GA2OX1	K,A	1E-123
	Ciunshiu_m21399		K04125	GA2OX2	K,A	1E-137
	Ciunshiu_m21688		K04125	GA2OX4	K,A	7E-99
	Ciunshiu_m15147		K04125	GA2OX6	K,A	1E-102
	Ciunshiu_m05431		K04125	GA2OX8	K,A	1E-131
	Ciunshiu_m06387		K04125	GA2OX8	K,A	1E-101
	Ciunshiu_m06397		K04125	GA2OX8	K,A	1E-101
	Ciunshiu_m10333		K04125	GA2OX8	K,A	1E-102
Gibberellin 3-oxydase (GA3ox)		4				
	Ciunshiu_m09507		K04124	GA3OX1	K,A	1E-114
	Ciunshiu_m14603		K04124	GA3OX1	K,A	2E-90
	Ciunshiu_m24574		K04124	GA3OX2	K,A	1E-116
	Ciunshiu_m24575		K04124	GA3OX2	K,A	1E-146
Gibberellin 13-oxydase, putative (CYP714, GA13ox)		3				
	Ciunshiu_m02939		K20666	CYP714A1	A,U,K	0
	Ciunshiu_m18898		K20666	CYP714A1	A,U,K	1E-148
	Ciunshiu_m17079		K20666	CYP714C2	U	1E-168

C. Carotenoids and abscisic acid biosynthesis

Gene name	C. unshiu gene	No. genes	KO	Gene symbol	Evidence	*E*-value
1. Carotene biosynthesis						
Phytoene synthase (PSY)		3				
	Ciunshiu_m07474		K02291	PSY	K,A	1E-124
	Ciunshiu_m07478		K02291	PSY	K,A	1E-146
	Ciunshiu_m19356		K02291	PSY	K,A	0

Phytoene desaturase (PDS)	1				
Ciunshiu_m07366		K02293	PDS	K,A	0
15-cis-zeta-carotene isomerase (Z-ISO)	1				
Ciunshiu_m17859		K15744	Z-ISO	K,A	1E-154
Zeta-Carotene desaturase (ZDS)	3				
Ciunshiu_m09863		K00514	ZDS	K,A	0
Ciunshiu_m14069		K00514	ZDS	A	0
Ciunshiu_m02861		K00514	ZDS	A	1E-162
Carotenoid isomerase/prolycopene isomerase (CRTISO)	1				
Ciunshiu_m19131		K09835	CRTISO	K,A	0
Lycopene epsilon-cyclase (LCYE)	1				
Ciunshiu_m14806		K06444	LCYe, crtL2, LUT2	K,A	0
Lycopene beta-cyclase (LCYB)	2				
Ciunshiu_m13050		K06443	LCYb	K,A	0
Ciunshiu_m21164 [*)]		K06443	LCYb	A	1E-163
2. β,β-Xanthophyll biosynthesis and xanthophyll cycle					
Carotene epsilon-monooxygenase (CYP97C)	1				
Ciunshiu_m08834		K09837	CYP97C1, LUT1	K,A	0
Beta-carotene 3-hydroxylase (CYP97A)	1	betaCHX			
Ciunshiu_m13985		K15747	CYP97A3, LUT5	K,A	0
Beta-carotene 3-hydroxylase (CHYB)	2	betaCHX			
Ciunshiu_m02366		K15746	CHYB, HYb, crtZ	K,A	1E-118
Ciunshiu_m15738		K15746	CHYB, HYb, crtZ	K,A	1E-107
Zeaxanthin epoxidase (ZEP)	2				
Ciunshiu_m00036		K09838	ZEP, ABA1	K,A	0
Ciunshiu_m13962		K09838	ZEP, ABA1	A	0
Violaxanthin de-epoxidase (VDE)	1				
Ciunshiu_m23017		K09839	VDE1, NPQ1	K,A	0
Carotenoid cleavage dioxygenase (CCD)	7				
Ciunshiu_m02163		K09840	CCD1	A	0
Ciunshiu_m10339		K09840	CCD4	K,A	0
Ciunshiu_m11823		K09840	CCD4	A	1E-115
Ciunshiu_m11820		K09840	CCD4	K,A	1E-144
Ciunshiu_m05099		K09840	CCD7	A	0
Ciunshiu_m25463		K09840	CCD8	A	0
Ciunshiu_m25469		K09840	CCD8	A	0
3. Violaxanthin catabolism					
9-cis-epoxycarotenoid dioxygenase (NCED)	3				
Ciunshiu_m13415		K09840	NCED3	K,A	0
Ciunshiu_m21417		K09840	NCED5	K,A	0
Ciunshiu_m14561		K09840	NCED6	K,A	0
Neoxanthin synthase, putative (NXS)	1				
Ciunshiu_m21164		K14594	NXS, NSY	U	0
Xanthoxin dehydrogenase (ABA)	1				
Ciunshiu_m25984		K09841	ABA2	K,A	1E-131
Abscisic-aldehyde oxidase (AAO)	2				
Ciunshiu_m21108		K09842	AAO4	K,A	0
Ciunshiu_m21109		K09842	AAO4	K,A	0
Beta-carotene isomerase (D27)	1				
Ciunshiu_m10903		K17911	DWARF27	K,A	9E-56

*) Dually assigned to LCYB or NXS.

KO: KEGG orthology (http://www.genome.jp/kegg/ko.html).

Gene symbol: reffered from the symbol of orthologue of Arabidopsis or Uniprot.

Evidence: bases of estimation. K; deduced from KAAS, A; deduced from similarity to the Arabidopsis gene, U; deduced from similarity t

E -value: TBLASTX or BLASTX similarity score to the orthologue of Arabidopsis or Uniprot.
