# Supplementary material for: Draft Sequencing of the Heterozygous Diploid Genome of Satsuma (Citrus unshiu Marc.) Using a Hybrid Assembly Approach
Source: Front Genet. 2017 Dec 5;8:180. doi: 10.3389/fgene.2017.00180 (PMC5723288; doi:10.3389/fgene.2017.00180)
Supplement: Supplementary file 9 [file Image1.PDF]

Shimizu, T. et al (2017) Draft sequencing of the heterozygous diploid genome of Satsuma (*Citrus unshiu* Marc.) using a hybrid assembly approach

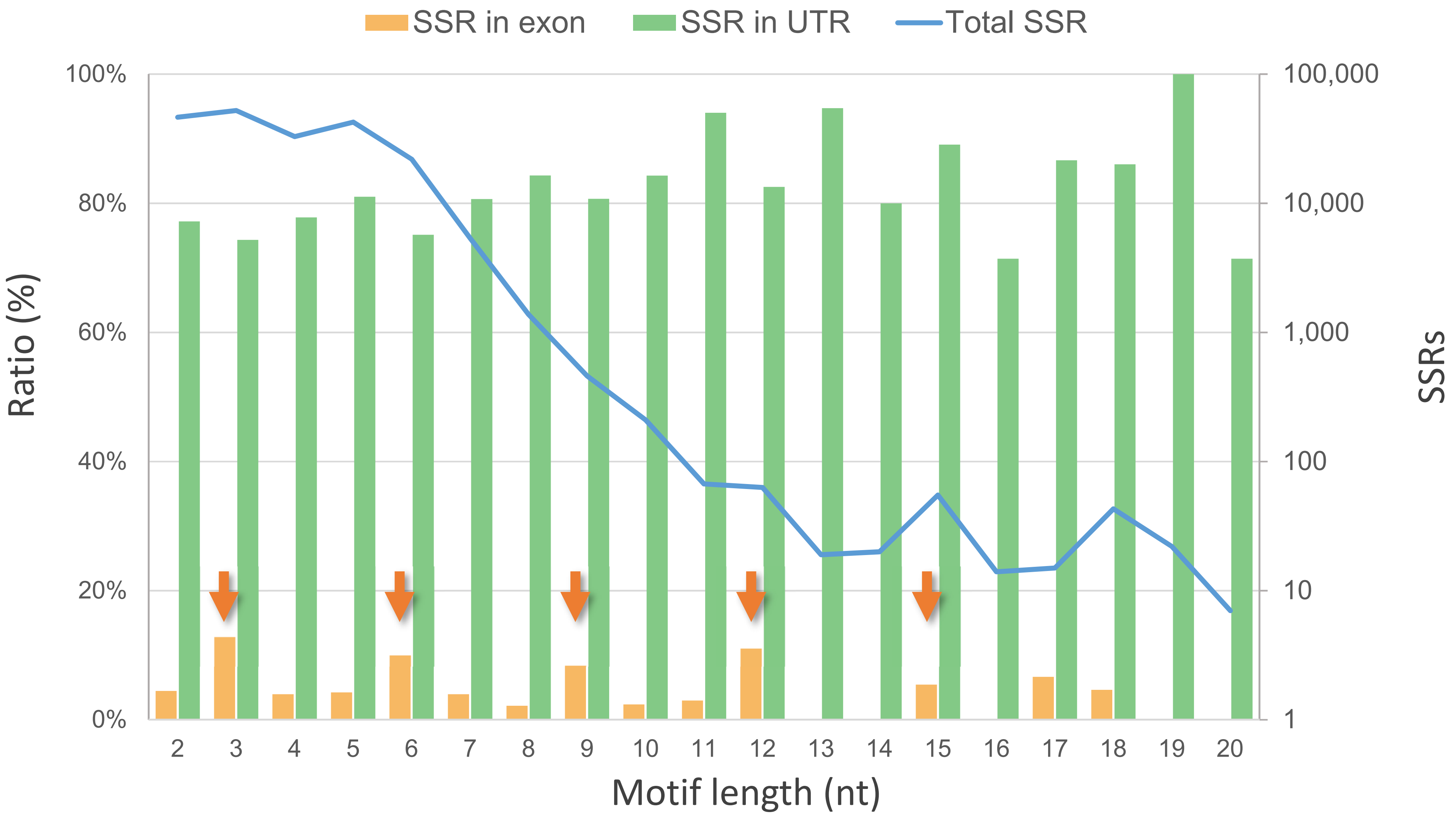

Supplemental Figure S1 Frequency distribution of the simple sequence repeat (SSR) motif length

Abscissa axis, motif length of SSRs (nucleotides); right ordinate axis, number of detected SSRs (blue line); and left ordinate axis, relative ratios of SSRs found in exons of the predicted protein coding genes (orange bar) or SSRs found in untranscribed region (UTR; green bar) to the total SSRs at each motif length. Red arrows indicate the increase in the ratio of SSRs in exons at triplet motifs.
