# Supplementary material for: Draft Sequencing of the Heterozygous Diploid Genome of Satsuma (Citrus unshiu Marc.) Using a Hybrid Assembly Approach
Source: Front Genet. 2017 Dec 5;8:180. doi: 10.3389/fgene.2017.00180 (PMC5723288; doi:10.3389/fgene.2017.00180)
Supplement: Supplementary file 10 [file Image2.PDF]

Shimizu, T. et al (2017) Draft sequencing of the heterozygous diploid genome of Satsuma (*Citrus unshiu* Marc.) using a hybrid assembly approach

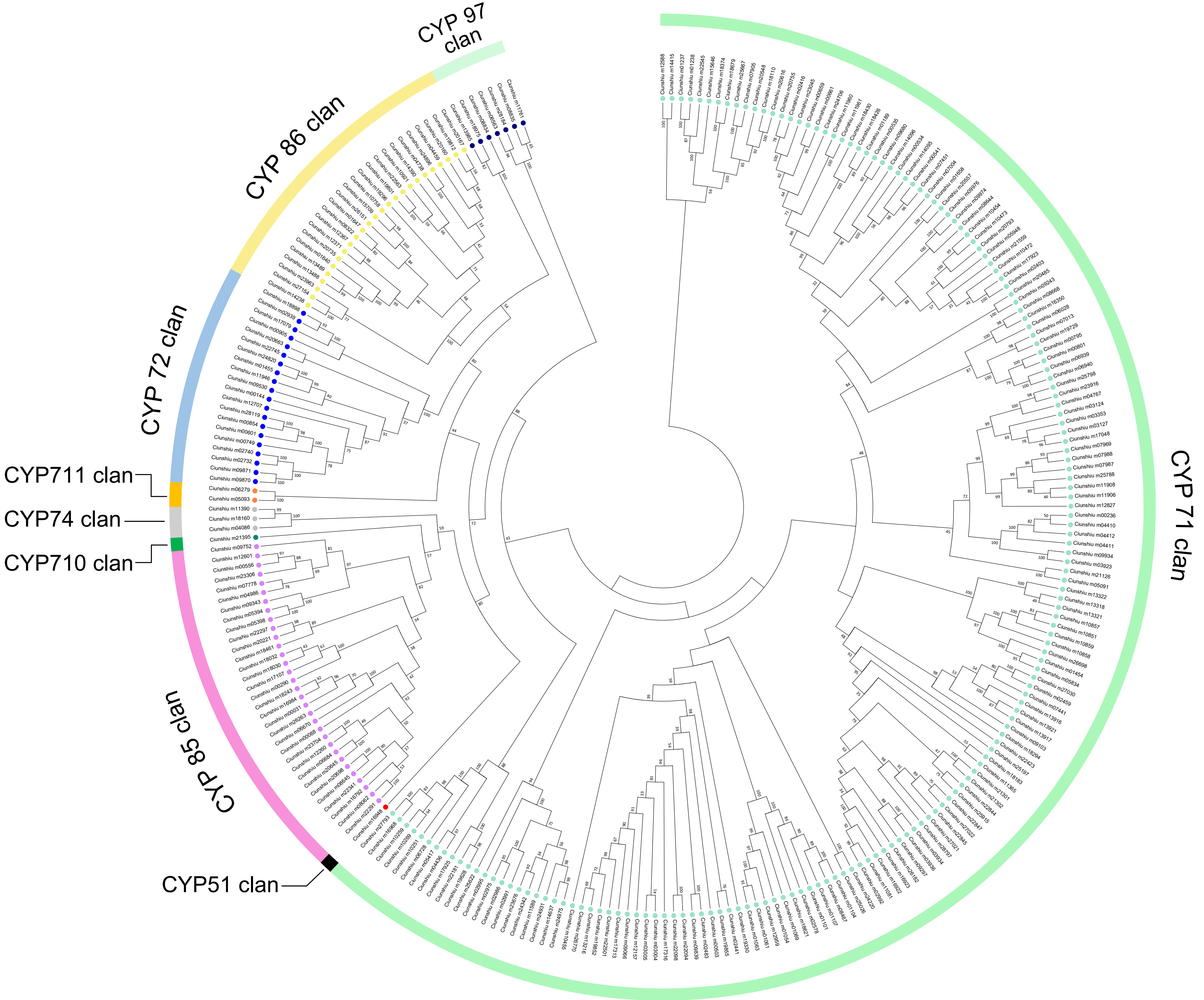

Supplemental Figure S2 Circular dendrogram of Satsuma P450 gene family

The dendrogram was constructed from the translated sequences of predicted protein by Neighbor-Joining method. The optimal tree with the sum of branch length = 85.14 is shown. The evolutionary distances were computed using the Poisson correction method and are in the units of the number of amino acid substitutions per site. The analysis involved 262 amino acid sequences. All ambiguous positions were removed for each sequence pair. There were a total of 4718 positions in the final dataset. Evolutionary analyses were conducted in MEGA7.
