# Supplementary material for: Draft Sequencing of the Heterozygous Diploid Genome of Satsuma (Citrus unshiu Marc.) Using a Hybrid Assembly Approach
Source: Front Genet. 2017 Dec 5;8:180. doi: 10.3389/fgene.2017.00180 (PMC5723288; doi:10.3389/fgene.2017.00180)
Supplement: Supplementary file 11 [file Image3.pdf]

Shimizu, T. et al (2017) Draft sequencing of the heterozygous diploid genome of Satsuma (*Citrus unshiu* Marc.) using a hybrid assembly approach

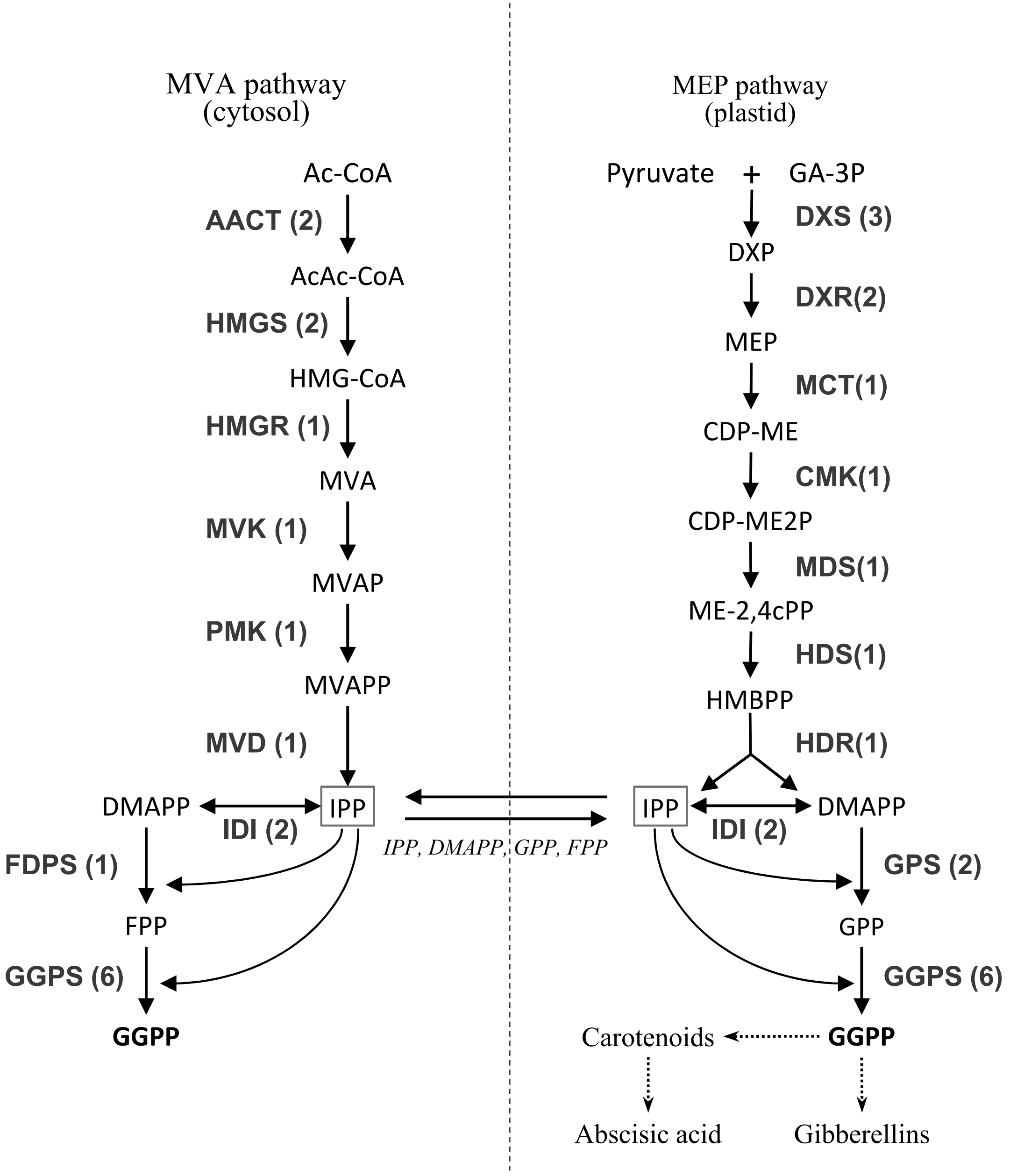

Supplemental Figure S3 Genes involved in the biosynthesis of isoprenoid (IPP) and geranylgeranyl diphosphate (GGPP) in Satsuma

MVA, mevalonate; Ac-CoA, acetyl-CoA; AACT, acetoacetyl-CoA (AcAc-CoA) C-acetyltransferase; HMGS, hydroxymethylglutaryl-CoA (HMG-CoA) synthase; HMGR, HMG-CoA reductase; MVK, mevalonate kinase; MVAP, mevalonate-5-phosphate; PMK, phosphomevalonate kinase, MVAPP, mevalonate-5-diphosphate; MVD, diphosphomevalonate decarboxylase; IDI, isopentenyl diphosphate (IPP) delta-isomerase; DMAPP, dimethylallyl diphosphate; FDPS, farnesyl diphosphate (FPP) synthase; GGPS, geranylgeranyl diphosphate (GGPP) synthase; MEP, 2-C-Methyl-D-erythritol 4-phosphate; GA-3P, D-glyceraldehyde 3-phosphate; DXS, 1-deoxy-D-xylulose-5-phosphate (DXP) synthase; DXR, DXP reductoisomerase; MCT, MEP cytidylyltransferase; CDP-ME, 4-(cytidine 5′-diphospho)-2-C-methyl-D-erythritol; CMK, CDP-ME kinase; CDP-ME2P, 2-phospho-4-(cytidine 5′-phospho)-2-C-methyl-D-erythritol; MCS, 2-C-methyl-D-erythritol 2,4-cyclodiphosphate (ME-2,4cPP) synthase; HDS, 1-hydroxy-2-methyl-2-butenyl 4-diphosphate (HMBPP) synthase; HDR, HMBPP reductase; GPS, geranyl diphosphate (GPP) synthase; and GGPP, geranylgeranyl diphosphate. Numbers in parentheses represent the number of each gene detected.
